# Bioprinting of inorganic-biomaterial/neural-stem-cell constructs for multiple tissue regeneration and functional recovery

**DOI:** 10.1093/nsr/nwae035

**Published:** 2024-01-25

**Authors:** Hongjian Zhang, Chen Qin, Zhe Shi, Jianmin Xue, Jianxin Hao, Jinzhou Huang, Lin Du, Hongxu Lu, Chengtie Wu

**Affiliations:** State Key Laboratory of High Performance Ceramics and Superfine Microstructure, Shanghai Institute of Ceramics, Chinese Academy of Sciences, Shanghai 200050, China; Center of Materials Science and Optoelectronics Engineering, University of Chinese Academy of Sciences, Beijing 100049, China; State Key Laboratory of High Performance Ceramics and Superfine Microstructure, Shanghai Institute of Ceramics, Chinese Academy of Sciences, Shanghai 200050, China; State Key Laboratory of High Performance Ceramics and Superfine Microstructure, Shanghai Institute of Ceramics, Chinese Academy of Sciences, Shanghai 200050, China; State Key Laboratory of High Performance Ceramics and Superfine Microstructure, Shanghai Institute of Ceramics, Chinese Academy of Sciences, Shanghai 200050, China; State Key Laboratory of High Performance Ceramics and Superfine Microstructure, Shanghai Institute of Ceramics, Chinese Academy of Sciences, Shanghai 200050, China; Center of Materials Science and Optoelectronics Engineering, University of Chinese Academy of Sciences, Beijing 100049, China; State Key Laboratory of High Performance Ceramics and Superfine Microstructure, Shanghai Institute of Ceramics, Chinese Academy of Sciences, Shanghai 200050, China; Center of Materials Science and Optoelectronics Engineering, University of Chinese Academy of Sciences, Beijing 100049, China; State Key Laboratory of High Performance Ceramics and Superfine Microstructure, Shanghai Institute of Ceramics, Chinese Academy of Sciences, Shanghai 200050, China; Center of Materials Science and Optoelectronics Engineering, University of Chinese Academy of Sciences, Beijing 100049, China; State Key Laboratory of High Performance Ceramics and Superfine Microstructure, Shanghai Institute of Ceramics, Chinese Academy of Sciences, Shanghai 200050, China; Center of Materials Science and Optoelectronics Engineering, University of Chinese Academy of Sciences, Beijing 100049, China; State Key Laboratory of High Performance Ceramics and Superfine Microstructure, Shanghai Institute of Ceramics, Chinese Academy of Sciences, Shanghai 200050, China; Center of Materials Science and Optoelectronics Engineering, University of Chinese Academy of Sciences, Beijing 100049, China

**Keywords:** 3D bioprinting, neural stem cells, inorganic biomaterials, neural constructs, multiple tissue regeneration

## Abstract

Tissue regeneration is a complicated process that relies on the coordinated effort of the nervous, vascular and immune systems. While the nervous system plays a crucial role in tissue regeneration, current tissue engineering approaches mainly focus on restoring the function of injury-related cells, neglecting the guidance provided by nerves. This has led to unsatisfactory therapeutic outcomes. Herein, we propose a new generation of engineered neural constructs from the perspective of neural induction, which offers a versatile platform for promoting multiple tissue regeneration. Specifically, neural constructs consist of inorganic biomaterials and neural stem cells (NSCs), where the inorganic biomaterials endows NSCs with enhanced biological activities including proliferation and neural differentiation. Through animal experiments, we show the effectiveness of neural constructs in repairing central nervous system injuries with function recovery. More importantly, neural constructs also stimulate osteogenesis, angiogenesis and neuromuscular junction formation, thus promoting the regeneration of bone and skeletal muscle, exhibiting its versatile therapeutic performance. These findings suggest that the inorganic-biomaterial/NSC-based neural platform represents a promising avenue for inducing the regeneration and function recovery of varying tissues and organs.

## Teaser

A new concept that developing an engineered inorganic biomaterials/neural stem cells (NSCs)-based neural platform to resolve the dilemma of multiple tissue regeneration with functions recovery in regenerative medicine is proposed.

## INTRODUCTION

The human body is a highly complex organism, being composed of various tissues and organs that work in harmony, necessitating the tight interaction of multiple systems and signal pathways [1]. The nervous system plays a leading role in regulating and controlling different physiological activities [2]. The central nervous system maintains the body's internal homeostasis and governs the movement of the musculoskeletal system [3]. Peripheral nerves are abundantly innervated in various tissues and actively participate in tissue development and metabolism [4]. In the context of bone development, sensory nerves determine the formation of primary/secondary ossification centers and later vascularization when bones grow [5]. Furthermore, neurotransmitters and neurotrophic factor signals from peripheral nerves also regulate bone metabolism and regeneration [6]. Nerves also direct the maturation and contraction of skeletal muscles through the formation of neuromuscular junctions (NMJs) [7]. Consequently, the nervous system acts as a commander, orchestrating the homeostasis and physiological functions of multiple tissues and organs within the human body.

Tissue regeneration is a sophisticated physiological process that requires the guidance of the nervous system and the synergistic action of other systems. Peripheral nerves first collect the ‘tissue-repairing signals’ from the injured areas and then send them to the central nervous system, thus initiating the tissue-repair procedures [8]. Additionally, various cells, cytokines and signals are involved in tissue regeneration, with nerves providing regulatory signals and neurotransmitters to tissue-resident cells [9]. Unfortunately, current repair strategies mainly focus on designing biomaterials to improve the biological activities of damaged tissue-resident cells or provide exogenous tissue-related cells to facilitate tissue regeneration, neglecting the crucial roles of nerves in tissue repair. Hence, a neural-induction-based tissue engineering strategy should be fully appreciated and explored, as it may offer a new approach for multiple tissue regeneration.

Neural stem cells (NSCs) reside in the nervous system with self-renewable and multidirectional differentiation properties and have the ability to reconstruct neural components [10]. However, their practical use has been limited by their fragility, sensitivity and uncontrolled differentiation properties [11]. Recently, inorganic materials have attracted more attention owing to their superior performance in promoting angiogenesis, osteogenesis, adipogenesis, wound healing and myocardial regeneration [12]. These materials release multiple bioactive ions (Ca, Mg, Sr, Zn, Si, Li, etc.), creating suitable ionic micro-environments to regulate the biological behaviors of various tissue-related cells [13]. Moreover, certain ions (e.g. Li, Ca and Si) have been found to have beneficial neuro-modulation effects, including neuroprotection, neuronal differentiation and neuron maturation [14–17]. For example, Li has neuroprotective effects as it inhibits the expression of apoptosis-related proteins p53 and Bax [18]. Li can also promote neuronal differentiation and maturation through inhibiting the activity of glycogen synthase kinase-3 (GSK-3β) [19]. Besides, Ca ions have the ability to promote the survival and differentiation of NSCs via activating the calcium signal pathway [15,20]. Moreover, it is reported that silicon (Si) ions could induce the axon outgrowth and secretion of neurotransmitters of sensory neurons, exhibited suitable neurogenesis activities [21]. Therefore, the preparation of inorganic-biomaterial/NSC-based neural constructs may serve as a promising approach for tissue regeneration with broad-spectrum application.

**Figure 1.**
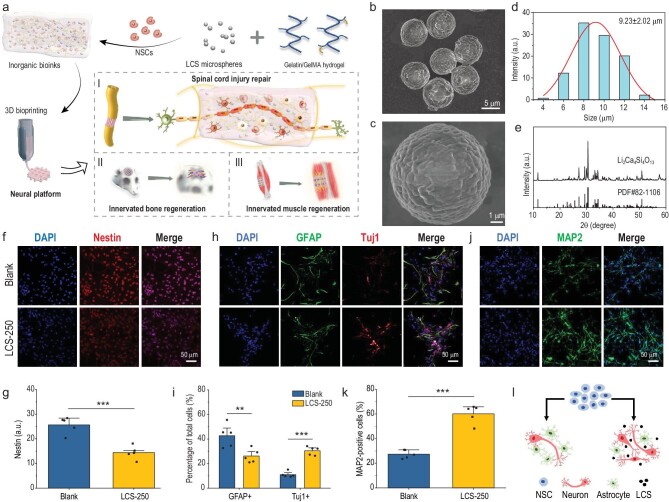
Schematic illustration of the inorganic-biomaterials-based neural platform and the characterization of the biological effects of LCS microspheres. (a) Schematic illustration of the preparation and application of an inorganic-biomaterials-based neural platform. Inorganic bioinks were composed of gelatin, gelatin methacryloyl (GelMA), Li-Ca-Si (LCS) microspheres and neural stem cells (NSCs). The 3D bioprinted neural constructs could serve as a versatile platform for promoting the regeneration of multiple tissues with functional recovery, including spinal cord injury repair, innervated bone regeneration and innervated muscle regeneration. (b and c) SEM images of LCS microspheres. (d) Size distribution of LCS microspheres. (e) X-ray diffraction (XRD) pattern of LCS microspheres. (f) Representative immunofluorescence staining images of Nestin proteins expression in NSCs after 5 days of culture. (g) Quantitative analysis of the mean fluorescence intensity of Nestin in Blank and LCS-250 groups (*n* = 5). (h) Representative immunofluorescence staining images of GFAP and Tuj1 proteins expression in NSCs after 5 days of culture. (i) Percentage of GFAP- and Tuj1-positive cells per field (*n* = 5). (j) Representative immunofluorescence staining images of MAP2 proteins after 10 days of culture. (k) Percentage of MAP2-positive cells per field (*n* = 5). (l) Schematic depiction of the stimulatory effects of LCS microspheres on the neural differentiation of NSCs. LCS bioceramic microspheres significantly stimulated the neuronal differentiation of NSCs and neuron maturation. Data are presented as the mean value ± SD. *P < 0.05, **P < 0.01, ***P < 0.001.

Herein, we have proposed a type of inorganic-biomaterial/NSC-based neural construct for promoting the regeneration of multiple tissues and function recovery (Fig. 1a). Both Li-Ca-Si (LCS) bioceramic and NSCs were incorporated into the Gelatin/Gelatin methacryloyl (GG) hydrogels to form inorganic bioinks. The distribution and arrangement of hydrogel filaments were flexibly controlled to match the targeted tissues through the 3D bioprinting technique. LCS bioceramic significantly improved the survival, proliferation, neural differentiation and maturation of NSCs by activating the PI3K-AKT signal pathway. Using a complete spinal cord transection model, we investigated the therapeutic effects of 3D bioprinted neural constructs on central nervous system injuries. The neural constructs stimulated neurogenesis and recovered locomotor functions. Additionally, the neural constructs also have positive modulation effects on osteogenesis, angiogenesis and formation of neuromuscular junctions *in vitro*. Furthermore, we verified the potential capacity of neural constructs in both hard and soft tissue regeneration using cranial-bone-defect and volumetric-muscle-loss-injury models. Overall, 3D bioprinting of inorganic-biomaterial/NSC-based neural constructs is expected to be a new concept for promoting multiple tissue regeneration with functional recovery from the perspective of neural induction. It provides new insights for the design and preparation of next-generation tissue engineering biomaterials.

## RESULTS

### Biological effects of Li-Ca-Si microspheres on NSCs

Firstly, LCS microspheres were prepared via the sol-spray method. Briefly, Li, Ca and Si salt solutions were mixed to form sol and then sprayed by a spray granulator to generate microspheres. The scanning electron microscopy (SEM) images (Fig. 1b and c) show that LCS displayed a well-dispersed microsphere morphology without conspicuous aggregations. The average diameter of LCS microspheres was 9.23 ± 2.02 μm (Fig. 1d). The X-ray diffraction (XRD) pattern shows that all the diffraction peaks of LCS microspheres could be well indexed into the phase of Li_2_Ca_4_Si_4_O_13_ (PDF#82-1106) (Fig. 1e) [22].

Subsequently, the regulatory effects of LCS microspheres on the proliferation and neural differentiation behaviors of NSCs were investigated. Firstly, NSCs, identified by the typical marker Nestin (Fig. S1 in the online supplementary file), were cultured in a proliferation medium containing various contents of LCS microspheres. The live/dead staining and cell morphology images show that NSCs spread well with an elongated morphology and maintained high viabilities when the concentrations of LCS microspheres were <500 μg/mL. Excessive LCS (over 500 μg/mL) microspheres may cause cytotoxicity due to burst release of ions (Fig. S2 and Table S1). Then, NSCs were cultured in a differentiation medium supplemented with 0, 100, 250 and 500 μg/mL of LCS for 5 days to explore their neural differentiation behaviors. Genes expression of neural-related markers, including Nestin, glial fibrillary acid protein (GFAP), β-III tubulin (Tuj1) and microtubule-associated protein 2 (MAP2) were assessed via real-time quantitative polymerase chain reaction (RT-qPCR) assay. As a stemness marker, Nestin is highly expressed in undifferentiated NSCs and decreased during the differentiation progress. GFAP is a typical marker of astrocytes, while Tuj1 is the microtubule protein of early neurons. MAP2 is a phosphorus protein that is the main component of neuronal filaments and represents a marker of mature neurons [23]. The Nestin expression level in LCS-microsphere-containing groups was significantly lower than in the Blank group, suggesting that LCS microspheres could reduce the stemness of NSCs (Fig. S3a). However, the expression level of GFAP in all groups had no obvious differences. Interestingly, the neuron-related marker Tuj1 and MAP2 expression level in LCS-microsphere-treated groups were higher than in the Blank group, especially in the LCS-250 group (NSCs cultured with 250 μg/mL of LCS microspheres) (Fig. S3b–d). The above results verified that LCS microspheres reduce the stemness of NSCs and induce NSCs to differentiate into mature neurons instead of astrocytes.

Furthermore, the relative expression of neural-specific proteins in the LCS-250 group was assessed. Fluorescence images and the corresponding statistical analysis results show that the intensity of Nestin in the LCS-250 group was significantly lower than in the Blank group, which was in accordance with the gene expression results (Fig. 1f and g). The images from the double immunofluorescence staining of GFAP and Tuj1 show that the GFAP-positive cells per field in the LCS-250 group were slightly less than in the Blank group, while the Tuj1-positive cells per field showed a remarkable reverse trend (Fig. 1h). Quantitatively, the average percentages (normalized to nuclei) of GFAP- and Tuj1-positive cells per field in the Blank group were 42.67 ± 9.32% and 10.98 ± 2.34%, respectively, showing that NSCs tended to differentiate into astrocytes more. In contrast, after being treated with LCS microspheres, 26.13 ± 5.57% of NSCs differentiated into GFAP-positive astrocytes, and 30.46 ± 3.45% of them differentiated into Tuj1-positive neurons (Fig. 1i). Overall, the proportion of astrocytes was reduced, while the proportion of neurons was significantly increased under the treatment of LCS microspheres with a concentration of 250 μg/mL. Moreover, as shown in Fig. 1j, after being cultured for 10 days, many more MAP2-positive cells were observed in the LCS-250 group than in the Blank group. The later mature neurons marker MAP2 showed a ∼33% increase under the treatment of LCS microspheres (Fig. 1k). LCS microspheres could significantly stimulate NSCs to more likely differentiate into neurons instead of astrocytes, and promote their further maturation (Fig. 1l).

### Preparation and characterization of LCS-microsphere-based neural constructs both *in vitro* and *in vivo*

**Figure 2.**
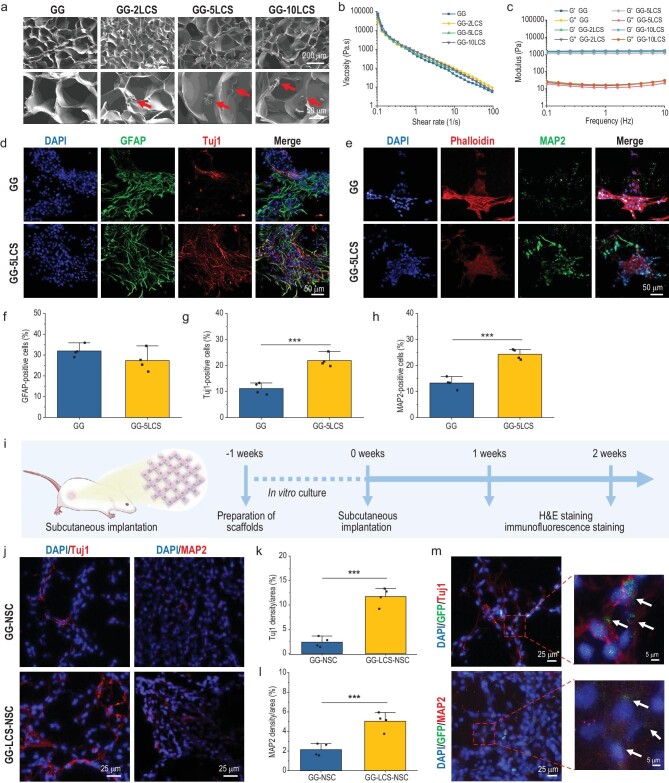
Characterization of the neuronal differentiation activity of 3D bioprinted neural constructs *in vitro* and *in vivo*. (a) SEM images of GG, GG-2LCS, GG-5LCS and GG-10LCS hydrogel inks. Red arrows indicate the incorporated LCS microspheres. (b) The shear-thinning properties of the hydrogel inks added with different contents of LCS. (c) The storage modulus (G′) and loss modulus (G′′) of these hydrogel inks with a frequency range of 0.1 to 10 Hz. (d) Representative double-immunofluorescence staining images of GFAP and Tuj1 of NSCs within GG and GG-5LCS constructs after 10 days of culture. (e) Representative immunofluorescence staining images of mature neuron marker MAP2 after 14 days of culture. (f–h) The percentage (normalized to nuclei) of GFAP- (f), Tuj1- (g) and MAP2- (h) positive cells per field in GG and GG-5LCS groups after (*n* = 4). (i) Schematic diagram of the procedure of subcutaneous implantation. (j) Representative immunofluorescence staining images of the neuronal markers Tuj1 and MAP2 in the constructs, 14 days post-implantation. (k and l) The quantitative analysis of Tuj1- and MAP2-positive areas per field *in vivo* (*n* = 4). (m) Double-immunofluorescence staining of GFP/Tuj1 and GFP/MAP2 demonstrated the survival, neuronal differentiation and maturation of exogenous NSCs within the constructs after being implanted for 14 days. White arrows indicate the co-localization. NSCs within the 3D bioprinted neural constructs exhibited superior survival, neuronal differentiation and neuron maturation activities under the stimulation of LCS microspheres both *in vitro* and *in vivo*. Data are presented as the mean value ± SD. *P < 0.05, **P < 0.01, ***P < 0.001.

Encouraged by the superior regulation effects of LCS microspheres on the neuronal differentiation of NSCs, LCS microspheres were incorporated into hydrogels to prepare bioinks. Gelatin (2 wt%) with reversible thermo-sensitive properties and GelMA (4 wt%) with thermo-sensitive and photo-crosslinking properties were used as the fundamental hydrogel matrix [24]. Due to its temperature-sensitive properties, gelatin acted as sacrificial phases to gradually dissolve from the constructs during the culture periods, leaving behind macro-porous interconnected networks (Fig. S4). The porous structures could not only facilitate the transportation of oxygen and nutrients but also provide a desirable 3D matrix environment for supporting the adhesion and growth of encapsulated cells. Subsequently, four kinds of bioinks containing different contents (0%, 2%, 5% and 10% relative to GelMA mass) of LCS microspheres were prepared, which were denoted as GG, GG-2LCS, GG-5LCS and GG-10LCS, respectively. As shown in Fig. 2a, all bioinks possessed macro-porous network structures, and LCS microspheres were uniformly distributed in the wall of the hydrogel matrix. The incorporation of LCS microspheres had neglected effects on the pore size of bioinks (Fig. S5). Rheological analysis shows that all bioinks exhibit suitable shear-thinning behaviors (Fig. 2b). Storage modulus (G′) was always larger than the loss modulus (G″) of all bioinks with a frequency range of 0.1 to 10 Hz (Fig. 2c). Furthermore, all bioinks showed favorable swelling behaviors, and the addition of LCS microspheres could slightly reduce the swelling ratio (Fig. S6). These results indicate that LCS-microsphere-laden bioinks exhibited satisfactory rheological behaviors, suitable for the following 3D bioprinting progress.

Subsequently, NSCs were added into the bioinks, and then deposited into the platform in a layer-by-layer fashion, followed by being crosslinked via blue light. All neural constructs maintained good shape integrity and the incorporation of LCS bioceramic barely affected the diameter of extruded filaments (Fig. S7). Subsequently, the viability of encapsulated NSCs was assessed by live/dead staining assay after culture for 1, 7 and 14 days. Cells in all groups survived well, and almost no red fluorescence (dead cells) could be observed in the whole culture period (Fig. S8). The proliferation rates of NSCs within the constructs were evaluated by the 5-ethynyl-2′-deoxyuridine (EdU) staining kits. The fluorescence images and quantitative analysis results show that the GG-5LCS group possessed more proliferative cells than other groups (Fig. S9). After 10 days of culture, cytoskeleton staining results indicated that NSCs adhered to the surface of the constructs and spread well (Fig. S10). These results confirmed that the LCS-based bioinks had suitable biocompatibility with the growth of NSCs.

Then, the neural differentiation behaviors of NSCs within the bioprinted constructs were explored by RT-qPCR and immunofluorescence staining. The expression level of stemness gene Nestin was decreased, while the neuronal differentiation gene Tuj1 was up-regulated in the GG-5LCS group as compared with the GG group (Fig. S11a and b). However, the expression of GFAP showed no significant differences (Fig. S11c). Double-immunofluorescence staining of GFAP and Tuj1 was performed after 10 days of culture in the neural differentiation medium (Fig. 2d). Many more Tuj1-positive cells could be observed in the GG-5LCS group than in the GG group. Quantitative analysis results show that the average percentages (normalized to nuclei) of Tuj1-positive cells per field in GG and GG-5LCS groups were 11.12 ± 2.2% and 21.89 ± 2.45%, respectively (Fig. 2g). In contrast, the percentage of GFAP-positive cells in these groups exhibited no significant differences (Fig. 2f). After 14 days of culture, a significantly higher ratio of MAP2-positive cells was observed in the GG-5LCS group (Fig. 2e and h). The introduction of 5% LCS microspheres significantly augmented the proliferation, neuronal differentiation and later maturation activities of NSCs within the 3D bioprinted constructs *in vitro*, while this content was used for all subsequent experiments.

In addition, it is of great significance to determine whether exogenous NSCs can survive and maintain their neuron differentiation capacity after implantation *in vivo*. Hence, we subcutaneously implanted the 3D bioprinted neural constructs (GG-NSC and GG-LCS-NSC) into the backs of sprague dawley (SD) rats for 7 and 14 days (Fig. 2i). The neural differentiation of NSCs was explored through histological staining. After 7 days of implantation, it could be observed that several blood scabs were distributed around the implanted constructs, indicating a mild inflammatory response (Fig. S12a). However, the implanted constructs integrated well with host tissues, accompanied by host blood vessel ingrowth after being implanted for 14 days. Hematoxylin and eosin (H&E) staining results further confirm that fibrous tissues infiltrated the internal part of the constructs (Fig. S12b). Subsequently, the immunofluorescence staining of Tuj1 and MAP2 showed that more neuron-specific proteins were expressed in the GG-LCS-NSC group than in the GG-NSC group (Fig. 2j). According to the quantitative analysis shown in Fig. 2k and l, the positive areas of Tuj1 and MAP2 in the GG-LCS-NSC group were 4.94-fold and 2.35-fold those in the GG-NSC group, respectively. Moreover, NSCs were labeled with green fluorescent proteins (GFPs) to monitor their fate *in vivo*. Surprisingly, GFP-labeled NSCs were co-localized with Tuj1 and MAP2 positive red fluorescence (Fig. 2m). This indicates that the exogenous NSCs (GFP-positive) within the bioprinted GG-LCS-NSC construct could differentiate into neurons and mature *in vivo*. These results showed that the LCS-microsphere-based bioinks endow NSCs with enhanced neural differentiation properties *in vivo*, which has high potential for central nervous system injury repairs and the regeneration of nerve-innervated tissues (bone, skeletal muscles, etc.).

### Signaling pathways underpinning the stimulatory effects of LCS microspheres on the neuronal differentiation of NSCs

**Figure 3.**
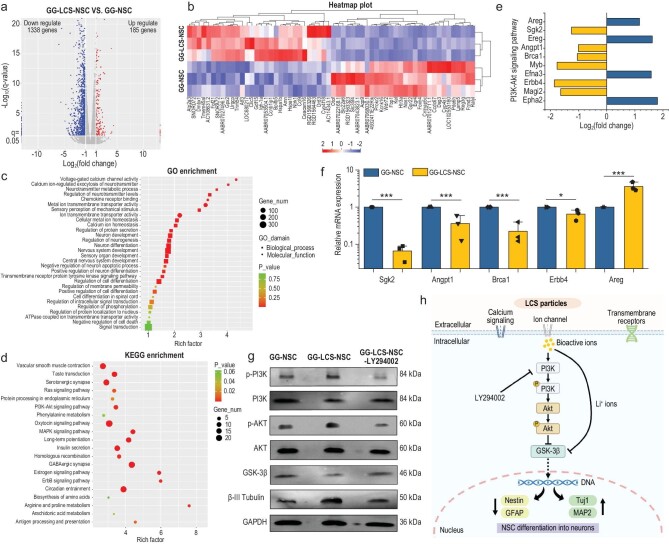
RNA-seq analysis of the mechanism of LCS microspheres for the neuronal differentiation of NSCs. (a) A volcano plot of differentially expressed genes (DEGs) of NSCs within the bioprinted GG-LCS-NSC and GG-NSC constructs, which were identified by RNA-sequencing with the cutoff: *p*-value < 0.05 and |log_2_ Fold Change| > 1. The 1338 down-regulated genes were marked in blue and the 185 up-regulated genes were marked in red. (b) Heat map plot of the top 30 differentially up-regulated and down-regulated genes of GG-LCS-NSC and GG-NSC groups. (c) Gene ontology (GO) enrichment of the biological function of DEGs. (d) Kyoto Encyclopedia of Genes and Genomes (KEGG) analysis of the signal pathway enriched by the DEGs. (e) Fold changes of the DEGs enriched in PI3K-AKT signal pathway. (f) RT-qPCR analysis results of the DEGs, which were involved in the PI3K-AKT signal pathway (*n* = 3). (g) Western blot analysis of the expression of proteins related to the PI3K-Akt signaling pathway and neuronal differentiation, including p-PI3K, PI3K, p-AKT, AKT, downstream target proteins GSK-3β and neuron marker β-III tubulin. (h) A schematic illustration of the potential underlying mechanism of LCS microspheres for the neuronal differentiation of NSCs within bioprinted constructs. LCS microspheres promote the neuronal differentiation of NSCs and neuron maturation within the bioprinted constructs via activating the PI3K-AKT signal pathway. Data are presented as the mean value ± SD. *P < 0.05, **P < 0.01, ***P < 0.001.

To elucidate the underlying mechanism of LCS microspheres stimulating the neural differentiation of NSCs, total gene expression of NSCs bioprinted within the GG-5LCS bioinks (GG-LCS-NSC) and NSCs bioprinted within the GG bioinks (GG-NSC) was detected by transcriptome sequencing (RNA-seq). The differentiated genes between the two groups were presented in a heat map, while up-expressed genes were marked in red, and down-expressed genes were marked in green (Fig. S13). A total of 1523 differentially expressed genes (DEGs) were determined by the cutoff: *p*-value < 0.05 and |log_2_ Fold Change| > 1. Compared with the GG-NSC group, the GG-LCS-NSC group had 185 up-regulated genes and 1338 down-regulated genes (Fig. 3a). Most DEGs, including 30 up-regulated genes and 30 down-regulated genes, are shown in Fig. 3b. Gene ontology (GO) analysis indicates that the DEGs were involved in the neural development and neurogenesis-related biological process, including neurotransmitter metabolic process, cellular metal ion homeostasis, neuron differentiation, nervous system development, negative regulation of the neuron apoptotic process and intracellular signal transduction (Fig. 3c). Moreover, the Kyoto Encyclopedia of Genes and Genomes (KEGG) analysis demonstrates that the PI3K-AKT signal pathway was significantly different between the GG-LCS-NSC and GG-NSC groups (Fig. 3d). Interestingly, several downstream targets of the PI3K-AKT signal pathway, including GSK-3β and γ-aminobutyric acid (GABA), play a significant role in neural development [25]. It is reported that calcium ions could activate the PI3K-AKT signal pathway to regulate the neural differentiation of NSCs [15]. Hence, we speculated that LCS microspheres may stimulate the neural differentiation of NSCs through activating the PI3K-AKT signal pathway.

Subsequently, whether LCS-containing bioinks activated the PI3K-AKT signal pathway was verified. Figure 3e shows the DEGs involved in the PI3K-AKT signal pathway, containing four up-regulated genes and six down-regulated genes in the GG-LCS-NSC group. Of these DEGs, five were randomly selected and detected by the RT-qPCR assay. Interestingly, the expression levels of the five genes were in accordance with the RNA-seq results (Fig. 3f). Subsequently, the protein expression levels of key markers involved in the PI3K-AKT signal pathway were assessed. LY294002, a chemical inhibitor, was added to inhibit the activity of PI3K kinase [26,27]. As shown in Fig. 3g, phosphorylated proteins p-PI3K and p-AKT were obviously up-regulated in the GG-LCS-NSC group compared to the GG-NSC group. However, the protein expression levels of p-PI3K and p-AKT in the GG-LCS-NSC group were remarkably reduced with the addition of LY294002. These results indicate that the LCS microspheres could activate the PI3K-AKT signal pathway. Furthermore, the expression levels of downstream target protein GSK-3β and neuron marker β-III tubulin were also examined. The results showed that the GG-LCS-NSC could significantly decrease GSK-3β expression but increase the β-III tubulin expression compared with the GG-NSC, and these effects can be counteracted by the addition of LY294002. From the above results, it can be concluded that the LCS microspheres promote the phosphorylation of p-PI3K and p-AKT proteins to activate the PI3K-AKT signal pathway, and inhibit the activity of GSK-3β, thereby stimulating the neural differentiation of NSCs (Fig. 3h).

### Neural constructs repairing the spinal cord injury with locomotor function recovery

**Figure 4.**
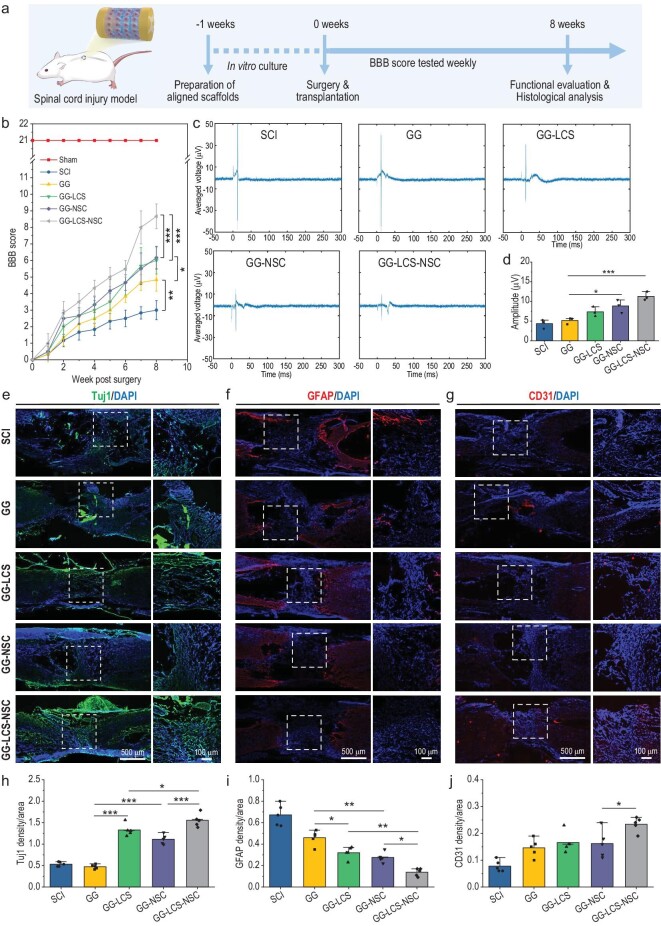
Therapeutic effects of 3D bioprinted neural constructs on the repair and functional recovery of spinal cord injury (SCI). (a) Schematic diagram of the procedure for SCI repair experiments. (b) The BBB score of SCI rats in each group. (c) Electrophysiological analysis of SCI rats in different groups after 8 weeks of post-surgery. (d) The average amplitudes of MEP of all groups after 8 weeks of surgery (*n* = 3). (e and f) Representative immunofluorescence staining images of Tuj1 (e) and GFAP (f) of the longitudinal sections of spinal cords, for evaluating neuronal regeneration. (g) Representative immunofluorescence staining images of CD31 of the longitudinal sections of spinal cords, for evaluating angiogenesis. (h–j) Statistical analysis of the Tuj1- (h), GFAP- (i) and CD31- (j) positive areas in the lesion regions (*n* = 5). 3D bioprinted GG-LCS-NSC neural constructs promoted the repairment and motor functional recovery of the spinal cord in a complete transection SCI model. Data are presented as the mean value ± SD. *P < 0.05, **P < 0.01, ***P < 0.001.

We used SD rats to build the spinal cord injury (SCI) model to explore the therapeutic effects of neural constructs *in vivo* [28]. All animals were divided into five groups: (i) Blank (non-treated); (ii) GG (3D printed GG constructs without LCS microspheres and NSCs); (iii) GG-LCS (3D printed GG-5LCS constructs without NSCs); (iv) GG-NSC (3D bioprinted NSC-laden constructs without LCS microspheres); (v) GG-LCS-NSC (3D bioprinted NSC-laden constructs containing LCS microspheres). Then, bioinks were deposited in an aligned architecture to form the constructs and cultured for 1 week in advance before filling in the gap of the complete transection of SCI (Fig. 4a and Fig. S14).

Firstly, the recovery of SCI rats’ hindlimb locomotor function was assessed every week according to the guideline of Basso Beattie-Bresnahan (BBB) scores [29,30]. Figure 4b shows that BBB scores of all groups increased over time, indicating that the locomotor function of all rats had varied degrees of recovery. After 8 weeks of surgery, rats in SCI groups were still in a paralysis state. Their ankle joint could move slightly, but the hindlimbs could only be dragged during crawling (BBB average score: 3 ± 0.63). In contrast, rats in the GG-LCS-NSC group showed better recovery of hindlimb locomotion, with the BBB scores at 8.67 ± 0.82, significantly higher than other groups. For instance, their ankle joint could extensively move, and the hindlimb could slightly support the body's weight during crawling (Fig. S15). Subsequently, an electrophysiological assay was performed to analyze the locomotor function recovery of all groups quantitatively (Fig. 4c) [31]. The average amplitudes of motor evoked potential (MEP) for the SCI, GG, GG-LCS, GG-NSC and GG-LCS-NSC groups were 4.37 ± 1.15, 5.16 ± 0.83, 7.37 ± 1.20, 8.86 ± 1.66 and 11.30 ± 1.10 μV, respectively (Fig. 4d). Overall, these results indicated that the GG-LCS-NSC group had the best capacity to recover the locomotor functions of SCI rats compared with the other groups.

Next, histological analysis and immunofluorescence staining were conducted to evaluate neural regeneration. From the gross images and H&E staining results shown in Fig. S16, a vast cavity was formed in the SCI group, which is the typical characteristic of traumatic spinal cord injury [23]. Interestingly, the implantation of bioprinted neural constructs obviously reduced the lesion cavity, and the GG-LCS-NSC group exhibited the best performance in this regard. Furthermore, immunofluorescence staining of neural-specific markers Tuj1, GFAP and neurofilaments (NFs) was used to evaluate neural regeneration in the injury areas. As shown in Fig. 4e, few Tuj1-positive neurons could be observed around the large cavity of lesion areas in the SCI group, while many more Tuj1-positive neurons were observed in the GG-LCS-NSC group. The quantitative analysis further confirms that the GG-LCS-NSC group had the highest density of neurons participating in the repair of spinal cord injuries (Fig. 4h). In contrast, the higher expression of GFAP (a marker of glial cells) in the SCI group represented the formation of glial scars in the lesion regions. In comparison, the bioprinted constructs group had much less expression of GFAP (Fig. 4f and i). Besides, the immunofluorescence staining of NFs further verified that the nerve fibers could grow throughout the transaction gaps under the guidance of bioprinted neural constructs (Fig. S17). These results demonstrate that the GG-LCS-NSC group exhibited the best performance of supporting neural growth and inhibiting glial scar formation. In addition, the spinal cord is densely vascularized via blood vessels and capillary networks, which play crucial roles in supporting neurite growth and spinal cord injury regeneration. Hence, the expression level of CD31 proteins in the lesion sites was assessed. As shown in Fig. 4g and j, the area of CD31-positive fluorescence in the GG-LCS-NSC group was more extensive than in other groups, indicating the superior capacity of the GG-LCS-NSC constructs to induce angiogenesis. Generally, these results show that the LCS-microsphere-based neural constructs could significantly promote neural regeneration and angiogenesis, thereby repairing the damaged spinal cord with functional recovery.

### Neural constructs modulating osteogenesis, angiogenesis and NMJs formation *in vitro*

As the central system of the human body, nerves are widely distributed in many tissues and organs and actively regulate non-neural cell growth, development and metabolism [32]. For example, neural cells actively participate in osteogenesis by secreting numerous neurotransmitters and neuropeptides [33]. Besides, owing to the parallel distribution of nerves and vessels, neural cells and endothelial cells also have a close interaction with each other and share the same signal pathways [34]. Moreover, nerves also control the contraction behaviors of skeletal muscles through NMJs [35]. Hence, it is exciting and necessary to explore the effects of neural constructs on tissue-related cells, which could provide a solid foundation for the further application of neural constructs on the repairment of nerve-innervated tissues.

Firstly, the pro-osteogenesis capacity of the bioprinted constructs was detected by indirect co-culture with bone marrow mesenchymal stem cells (BMSCs) with a transwell method (Fig. S18a and b). The migration, protein expression and mineralization deposition activities were assessed. Transwell migration images and statistical analysis show that more migrated BMSCs were observed in the GG-LCS-NSC group than in other groups (Fig. S18c and g). After co-culture for 5 days, the expression levels of osteogenesis-related proteins BSP and OCN were investigated (Fig. S18d). The intensities of BSP and OCN proteins in the GG-LCS-NSC group were significantly higher than those in other groups, suggesting that the LCS-microsphere-augmented neural constructs could stimulate the osteogenic differentiation of BMSCs (Fig. S18j and k). Subsequently, the alkaline phosphatase (ALP) activity and calcium nodule deposition ability was assessed (Fig. S18e and f). Significantly higher levels of ALP activity and mineralization nodules were observed in the GG-LCS and GG-LCS-NSC groups compared with the GG and GG-NSC groups (Fig. S18h and i). GG-LCS exhibited better performance on osteogenesis than the GG group, indicating the superior osteogenic activity of the LCS bioceramic. Overall, the 3D bioprinted GG-LCS-NSC neural constructs have remarkable osteogenesis capacities, possessing high potential in bone tissue regeneration.

Secondly, the angiogenic properties of bioprinted constructs were assessed by transwell migration, tube formation and immunofluorescence staining. Human umbilical vein endothelial cells (HUVECs) were co-cultured with the bioprinted constructs in transwell chambers (Fig. S19a and b). The migration activity of HUVECs in the GG-LCS-NSC group was obviously more facilitated than in other groups (Fig. S19c and f). Besides, the tube formation assay confirms that the number of junctions and meshes in the GG-LCS, GG-NSC and GG-LCS-NSC groups was obviously higher than in the GG group, among which GG-LCS-NSC had the best capacity to promote tube formation (Fig. S19d). Quantitatively, the number of junctions and meshes in the GG-LCS-NSC group was 5.48-fold and 9.79-fold higher than in the GG group (Fig. S19g and h). Furthermore, the immunofluorescence staining of angiogenic proteins (CD31 and VEGF) and the corresponding statistical analysis show that the bioprinted constructs containing LCS microspheres possessed the best properties of stimulating angiogenesis (Fig. S19e, i and j).

Finally, the ability of bioprinted neural constructs to form NMJs *in vitro* was investigated by co-culture with rat muscle cells (L6 cells) (Fig. S20a). Double-immunofluorescence staining of neuron marker Tuj1 and acetylcholine receptor (AchRs) was performed after co-culture for 5 days. AchRs clusters were closely contacted with Tuj1-positive neurons, indicating the successful formation of NMJs *in vitro* (Fig. S20b). More AchRs clusters were observed in the GG-LCS-NSC-L6 group than in the GG-NSC-L6 group. Quantitatively, the number of NMJs (Tuj1^+^/AchRs^+^) in the GG-LCS-NSC-L6 group was 2.75-fold higher than that in the GG-NSC-L6 group (Fig. S20c). Thus, neural constructs also exhibited great potential for skeletal muscle regeneration.

### Neural constructs promoting cranial bone regeneration with innervation and vascularization

**Figure 5.**
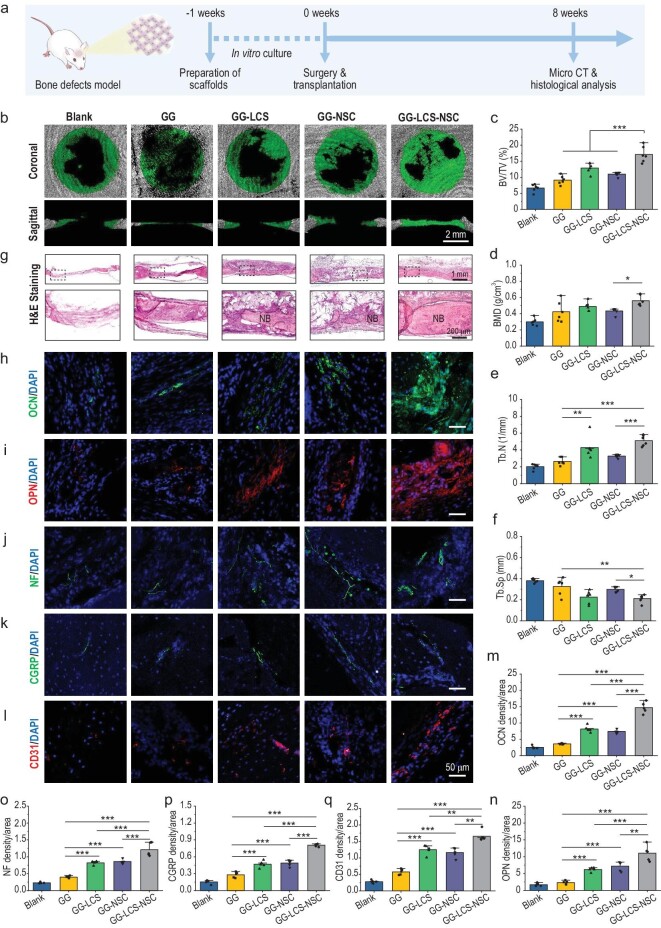
3D bioprinted neural constructs promoting bone regeneration in a cranial defect model. (a) A schematic diagram of the procedure for bone regeneration experiments. (b) Representative micro-CT reconstruction images of the bone defect regions in both the coronal and sagittal view. (c–f) Statistical analysis of the BV/TV (c), BMD (d), Tb.N (e) and Tb.Sp (f) values of regenerated bones (*n* = 6). (g) Microscope images of the H&E staining of the regenerated bone tissues at 8 weeks of implantation. (h and i) Representative images of osteogenic marker OCN (h) and OPN (i) for evaluating bone formation. (j and k) Representative images of neural marker NF (j) and CGRP (k) for evaluating innervation. (l) Representative images of angiogenic marker CD31 for evaluating vascularization. (m–q) Statistical analysis of the OCN (m), OPN (n), NF (o), CGRP (p) and CD31 (q) positive areas in the bone defect regions (*n* = 5). 3D bioprinted GG-LCS-NSC neural constructs significantly promoted bone regeneration with innervation and vascularization under the synergistic effects of NSCs and LCS bioceramic. Data are presented as the mean value ± SD. *P < 0.05, **P < 0.01, ***P < 0.001.

Encouraged by the satisfactory capacity of 3D bioprinted neural constructs on stimulating osteogenesis and angiogenesis *in vitro*, we further assessed the ability of bioprinted neural constructs on promoting bone formation *in vivo*. Cranial bone defect models were established with SD rats, and then the constructs (GG, GG-LCS, GG-NSC and GG-LCS-NSC) were implanted into the defects (Fig. S21). After 8 weeks post-implantation, cranial bone tissues were collected for micro-computed tomography (micro-CT) and histological analysis (Fig. 5a). Micro-CT images in coronal and sagittal views show that few newly formed bones were found in the Blank and GG groups, while a mass of new bone distributed from the margin to the center of defect regions was found in the GG-LCS-NSC group (Fig. 5b). Quantitative analysis suggests that the bone volume/total volume (BV/TV) value in the GG-LCS-NSC group (17.13 ± 2.51%) was 1.33-fold higher than in the GG-LCS group (12.90 ± 1.58%), and 1.56-fold more elevated than in the GG-NSC group (10.98 ± 0.66%) (Fig. 5c). It is worth noting that the GG-LCS had better effects on bone formation than the GG-NSC, indicating that bioceramic promotes osteogenesis through releasing bioactive ions. The quantification of bone mineral density (BMD), trabecular number (Tb.N) and trabecular separation/spacing (Tb.Sp) further demonstrates that GG-LCS-NSC had the best ability to promote bone formation (Fig. 5d–f). Subsequently, the histological analysis of the newly formed bone was conducted. As shown in Fig. 5g, the Blank group was filled with fibrous tissues, indicating a weak self-healing ability. In contrast, varied degrees of new bone could be observed in construct-implanted groups, while the GG-LCS-NSC group had the highest area of new bone and integrated well with surrounding host bones. Immunofluorescence staining images (Fig. 5h and i) and the corresponding statistical analysis (Fig. 5m and n) further demonstrate that the GG-LCS-NSC group had the highest expression area of bone marker OCN and OPN in the bone defect regions, which was in accordance with the micro-CT and H&E staining analysis.

Furthermore, as exhibited in Fig. 5j, much more NFs (cytoskeletal of neurons) were found in the GG-LCS-NSC group compared with other groups, indicating the suitable innervation of regenerated bone tissues [36]. It is worth noting that the major type of nerve that regulates bone healing are calcitonin gene-related peptide (CGRP) positive nerves [37]. Hence, the regeneration of CGRP-positive nerves was further detected. As expected, the GG-LCS-NSC group shows higher positive areas of CGRP immunofluorescence than the GG-LCS and GG-NSC groups (Fig. 5k). In addition, CD31, a marker of endothelial cells, was used to evaluate the vascularization behaviors. It can be found that much more CD31 positive fluorescence was expressed in the GG-LCS-NSC group, indicating the superior ability of GG-LCS-NSC constructs to promote vascularization (Fig. 5l). Furthermore, the quantitative analysis of NF, CGRP and CD31 positive areas also confirms that the GG-LCS-NSC had the best performance when it came to inducing innervation and vascularization (Fig. 5o–q). Generally, the superior performance of 3D bioprinted GG-LCS-NSC constructs on bone regeneration was mainly attributed to the incorporation of LCS microspheres and NSCs. On the one hand, bioactive ions released from LCS microspheres could directly promote osteogenesis and angiogenesis. On the other hand, LCS microspheres endow the incorporated NSCs with enhanced neuronal differentiation properties, which leads to better bone formation, vascularization and innervation.

### Neural constructs regenerating the skeletal muscle with neural integration and vascularization

**Figure 6.**
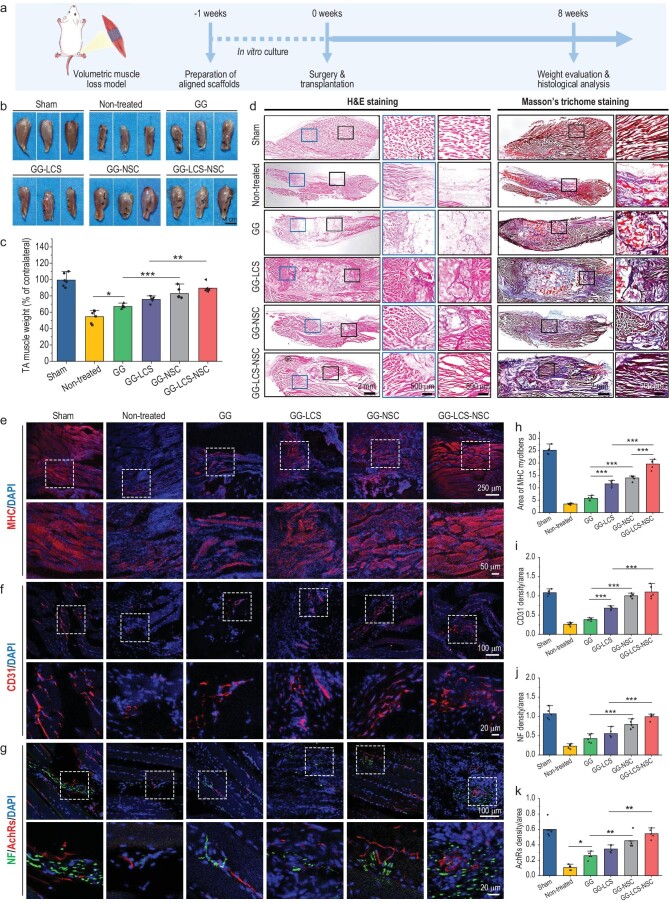
3D bioprinted neural constructs promoting muscle regeneration in a rat tibialis anterior (TA) muscle defect model. (a) A schematic diagram of the procedure for skeletal muscle regeneration experiments. (b) Representative optical images of the retrieved TA muscles after 8 weeks of implantation. (c) The weight of retrieved TA muscles (% of contralateral) after 8 weeks of implantation (*n* = 6). (d) Representative images of H&E staining and Masson's trichrome staining of the regenerated TA muscles. (e) Representative images of myogenic marker MHC, for evaluating the regeneration and maturation of newly formed myofibers. (f) Representative images of angiogenic marker CD31 for assessing vascularization. (g) Representative images of the double-immunofluorescence staining of AchRs and NF for evaluating neural integration. (h–k) Statistical analysis of the MHC (h), CD31 (i), NF (j) and AchRs (k) positive areas in the TA muscle defect regions (*n* = 5). 3D bioprinted GG-LCS-NSC neural constructs promoted myofiber formation and maturation, vascularization, and neural integration of the regenerated muscles. Data are presented as the mean value ± SD. *P < 0.05, **P < 0.01, ***P < 0.001.

Skeletal muscle is densely innervated by peripheral nerves through the formation of NMJs, which mediate the development, maturation and contraction behaviors of muscles [38]. Hence, enhancing the innervation of skeletal muscle defects is necessary but challenging for accelerating muscle regeneration and restoring its contraction ability. Under the stimulation of the LCS microspheres, the neural constructs showed superior performance with regard to the formation of NMJs *in vitro*, which encouraged us to explore its potential application in skeletal muscle repairs. Therefore, volumetric muscle loss (VML) defect models were established by cutting ∼40% of the tibialis anterior (TA) muscles of SD rats [39]. 3D bioprinted GG, GG-LCS, GG-NSC and GG-LCS-NSC constructs were fabricated and then implanted into the defect areas (Fig. 6a and Fig. S22). The Sham group, and Non-treated group with only defects, were used as controls. After 8 weeks of surgery, TA muscles were isolated from the body and their weights were recorded. The volumes of TA muscles from the GG-NSC and GG-LCS-NSC groups were close to those of the Sham group. In contrast, the Non-treated and GG groups exhibited obvious muscular atrophy (Fig. 6b). The TA muscle weight (percentage of contralateral) in the Sham, Non-treated, GG, GG-LCS, GG-NSC and GG-LCS-NSC groups was 99.41 ± 8.07%, 54.69 ± 7.78%, 67.03 ± 2.45%, 75.83 ± 3.59%, 83.01 ± 6.82% and 89.52 ± 5.29%, respectively (Fig. 6c). Overall, the GG-NSC and GG-LCS-NSC groups had the highest muscle weight as compared to other groups.

Subsequently, the myofiber morphology, vascularization and innervation of the regenerated muscles were evaluated by H&E, Masson's trichrome and immunofluorescence staining. The defect areas were filled with fibrous tissues. Few newly formed myofibers could be observed in the Non-treated and GG groups (Fig. 6d). In contrast, less fibrous tissue infiltration and highly organized new myofibers were found in the GG-LCS-NSC group, indicating the effective regeneration of muscles. Moreover, immunofluorescence staining of myosin heavy chain (MHC) was used to evaluate the regeneration of newly formed TA muscles. The areas of positive MHC expression in the Non-treated and GG groups were significantly less than those in GG-NSC and GG-LCS-NSC groups (Fig. 6e and h). Besides, it is worth noting that the newly formed myofibers in the GG-LCS-NSC group presented in a highly organized manner, indicating its maturation. Then, the vascularization ability of neural constructs was assessed. As shown in Fig. 6f and i, a larger CD31 positive area was found in the GG-LCS-NSC group than in other groups. Finally, the innervation and NMJs formation of the regenerated muscles were evaluated by double-immunofluorescence staining of NFs and AchRs. As exhibited in Fig. 6g, the co-localization of NF-positive nerves and AchRs could be observed in the defect regions, which indicated the successful neural integration and formation of NMJs *in vivo*. Notably, the highest expression of NF-positive nerves and AchRs was found in the GG-LCS-NSC group, showing the superior capacity for inducing NMJ formation (Fig. 6j and k). In summary, 3D bioprinted neural constructs containing LCS microspheres showed increased neural differentiation of NSC and NMJ formation capacity, resulting in enhanced skeletal muscle regeneration and neural integration. These results indicate that 3D bioprinting of inorganic-biomaterial-augmented neural constructs is a promising approach for skeletal muscle regeneration.

## DISCUSSION

The leading role of the nervous system in the human body means that reconstructing the neural components of injured sites is essential for ideal tissue regeneration and functional recovery [40]. Herein, we have developed inorganic-biomaterial/NSC-based constructs via 3D bioprinting technology, which could serve as a versatile platform for regenerating multiple tissues with functional recovery. The specific parameters (e.g. size, filament distance and filament arrangement) of the bioprinted constructs can be regulated by the printing set-up to mimic the topographical and physiological structures of injured tissues. The survival and differentiation of NSCs is favored in softer matrices, while softer matrices had poor printability and were not suitable for extrusion 3D bioprinting. Thus, we addressed the paradox of printability and biocompatibility through the combination of gelatin and GelMA. Sacrificial phase materials-Gelatin can aid the filament extrusion and maintains the structural stability during the fabrication process, and subsequently dissolved away from the constructs in the culture periods. More importantly, inorganic-biomaterial LCS microspheres were added into the hydrogels to serve as stimulatory agents to enhance the biological activities of NSCs. Owing to the multiple-ion release profiles of LCS microspheres, the bioinks could create a beneficial micro-environment for the survival of NSCs (Tables S1 and S2). Previous studies have demonstrated that Li ions have superior anti-apoptotic and neuroprotective properties. Specifically, Li ions could promote cytoprotective protein Bcl-2 expression while inhibiting the expression level of pro-apoptotic proteins p53 and Bax, thus enhancing cell viability. Furthermore, LCS microspheres promote the encapsulated-NSCs differentiated into neurons instead of astrocytes. RNA-seq and western blot results further confirmed that LCS microspheres obviously activated the PI3K-AKT signal pathway and decreased downstream target protein GSK-3β expression, resulting in increased expression of neuron marker β-III tubulin. However, with the addition of LY294002, the PI3K-AKT signal pathway was blocked, followed by the decreased expression of β-III tubulin. It is reported that calcium signaling could directly promote the neural differentiation of NSCs via the calcium ions channel [15]. Si ions could induce the axon outgrowth and secretion of neurotransmitters of sensory neurons, exhibiting suitable neurogenic activities [21]. Li ions have been verified that could directly inhibit the activity of GSK-3β, thus promoting neurogenesis. Therefore, it is reasonable to conclude that LCS microspheres can decrease the stemness while promoting the neuronal differentiation and maturation of NSCs, potentially through activating the PI3K-AKT signal pathway (Fig. 3h). In addition, it should be noted that due to the immunocompetence of animals, the survival and specific differentiation of exogenous NSCs *in vivo* have a great impact on their therapeutic efficiency. In the subcutaneous implantation experiments, the co-localization of GFP with neuron marker Tuj1 and neuron maturation marker MAP2 demonstrated the successful survival, neuronal differentiation and maturation of implanted exogenous NSCs, which is essential for *in vivo* therapeutic effects.

In the SCI repair experiments, the bioprinted neural constructs exhibited better performance with regard to the locomotion recovery of SCI rats after 8 weeks of implantation. The BBB score and MEP values in the GG-LCS-NSC group were 8.67 ± 0.82 and 11.30 ± 1.10 μV, respectively, significantly higher than the GG-LCS and GG-NSC groups. Histologically, increased neuronal regeneration, reduced lesion cavity and glial scar formation were also observed in the GG-LCS-NSC group. We inferred that incorporating LCS microspheres endows the encapsulated NSCs with enhanced neuronal differentiation capacity, which results in accelerated neuronal regeneration. Besides, bioactive ions released from the constructs could also activate the endogenous NSCs and differentiate into neurons to initiate endogenous neurogenesis. Generally, the bioprinted GG-LCS-NSC neural constructs significantly promoted SCI repair and functional recovery through the synergistic effects of exogenous and endogenous neurogenesis.

More excitingly, 3D bioprinted neural constructs also possessed superior capacities for osteogenesis and angiogenesis. It is known that neurotransmitters and neurotrophic factors from neural cells heavily contribute to bone formation and vascularization. We found that NGF and CGRP neurotransmitters could be detected in the medium of bioprinted neural constructs (Fig. S23). The concentration of CGRP was at a significantly higher level in the GG-LCS-NSC medium than in the GG-NSC medium. It is reported that NGF binds to its specific receptors TrK-A to stimulate the osteogenesis process [5,41]. It is also reported that CGRP can promote the osteogenic differentiation of BMSCs and mineralization deposition by activating the Wnt/β-catenin signal pathway [42]. CGRP can also promote the migration and tube formation of endothelial cells through binding to vascular receptors [43]. Hence, exogenous NSCs, especially under the stimulation of LCS microspheres, could secret multiple neurotransmitters and neurotrophic factors, which leads to enhanced osteogenesis and angiogenesis properties. Moreover, our groups have previously demonstrated that bioactive Li, Ca and Si ions also have a direct promotion effect on osteogenesis and angiogenesis. Consequently, 3D bioprinted GG-LCS-NSC neural constructs had the best performance when it came to stimulating innervation, vascularization and bone formation *in vivo*.

Surprisingly, many more AchRs clusters could be observed in the GG-LCS-NSC-L6 co-culture group compared with the GG-NSC-L6 group. It is known that the formation of NMJs depends on the activities of neural cells and muscle cells. As mentioned above, LCS microspheres significantly promoted the NSCs’ differentiation into neurons and maturation. The differentiated neural cells might enhance the biological activity of muscle cells through secreting neurotrophic factors. Besides, a recent study has demonstrated that Si ions can promote the myogenic differentiation of muscle cells [44]. Hence, we assume that under the presence of LCS microspheres, the biological activities of NSCs and L6 cells were enhanced, leading to increased NMJ numbers. In accordance with the *in vitro* experiments, 3D bioprinted GG-LCS-NSC neural constructs significantly restored the TA muscle weight, as well as enhanced the vascularization and neural integration of regenerated muscles.

Although this study presents a universal approach for multiple tissue regeneration and functional recovery, future studies still need to address several limitations. Firstly, we preliminarily verified the survival and neuronal differentiation behaviors of exogenous NSCs *in vivo* through subcutaneous implantation experiments. The long-term monitoring of the fate of exogenous NSCs is essential to evaluate their functions. Besides, owing to the multidirectional differentiation properties of NSCs, the respective effects of differentiated neurons and astrocytes on neural integration and tissue regeneration need to be further clarified. Secondly, several characterizations have not yet been done in this work due to the limited experimental equipment. The contractibility of the bioprinted GG-LCS-NSC-L6 co-culture construct *in vitro* and the tetanic force of the regenerated muscles *in vivo* have not been included. However, according to the formation of NMJs *in vivo* (Fig. 6), we still have reason to believe that the regenerated muscle was successfully integrated with host nerves and their function was restored to some extent. Finally, although the source of NSCs (derived from SD rats) was consistent with the species used in *in vivo* experiments, which avoided host immune response as far as possible, the inflammatory responses and foreign body reaction during the whole tissue regenerative process should be explored in the future.

## CONCLUSION

In summary, we have prepared an inorganic-biomaterial/NSC-based neural construct via 3D bioprinting technology, which could provide a versatile platform for promoting multiple tissue regeneration with functional recovery. The addition of LCS microspheres could improve the proliferation, neuronal differentiation and later maturation activities of encapsulated NSCs by activating the PI3K-AKT signal pathway. Furthermore, the neural constructs could not only accelerate the repair process of a central nervous system injury with functional recovery but also promote the regeneration of peripheral bone and skeletal muscle tissues, showing their versatile application. Overall, inorganic-biomaterial/NSC-based neural constructs provide a new way of thinking and a new approach to promoting tissue regeneration from the point of view of neural modulation, which will shed light on biomaterials design for regenerative medicine.

## MATERIALS AND METHODS

Detailed materials and methods are available in the supplementary data files.

## Supplementary Material

nwae035_Supplemental_File
