# Supplementary material for: Bioprinting of inorganic-biomaterial/neural-stem-cell constructs for multiple tissue regeneration and functional recovery
Source: Natl Sci Rev. 2024 Jan 25;11(4):nwae035. doi: 10.1093/nsr/nwae035 (PMC10924618; doi:10.1093/nsr/nwae035)
Supplement: nwae035_Supplemental_File [file nwae035_supplemental_file.pdf]

RESEARCH ARTICLE

MATERIALS SCIENCE

Bioprinting of Inorganic Biomaterials-Neural Stem Cells Constructs for Multiple Tissue Regeneration and Functional Recovery

Hongjian Zhang [1,2], Chen Qin [1], Zhe Shi [1], Jianmin Xue[1], Jianxin Hao [1,2], Jinzhou Huang [1,2], Lin Du [1,2], Hongxu Lu [1,2], Chengtie Wu[1,2]*.

[1] State Key Laboratory of High Performance Ceramics and Superfine Microstructure, Shanghai Institute of Ceramics, Chinese Academy of Sciences, Shanghai 200050, P. R. China.

[2] Center of Materials Science and Optoelectronics Engineering, University of Chinese Academy of Sciences, Beijing 100049, P. R. China.

***Corresponding author.** State Key Laboratory of High Performance Ceramics and Superfine Microstructure, Shanghai Institute of Ceramics, Chinese Academy of Sciences, Shanghai 200050, P. R. China.

*Email: chengtiewu@mail.sic.ac.cn

Supplementary Information-Materials and Methods

Synthesis and characterization of Li-Ca-Si (LCS) bioceramic

$Li_2Ca_4Si_4O_{13}$ bioceramic microspheres were synthesized using a sol-spray method. Firstly, Tetraethylorthosilicate (TEOS, Sinopharm, China), HNO_3 (Sinopharm, China), and deionized water were mixed uniformly with the molar ratio at 1:0.16:8. Secondly, $LiNO_3$ (Sinopharm, China) and Ca $(NO_3)_2$ 4H_2O (Sinopharm, China) were dropwise into the sol with the molar ratio of Li: Ca: Si at 1:2:2 and uniformly stirred for 1 h. Thirdly, the obtained sol was sprayed by the spray granulator (Shanghai Qiao Feng, China) to obtain the green bodies of microspheres, followed by sintered at 940 ℃ for 3 h with a heating rate at 2 ℃/min and sieved through 1000-mesh sieves to obtain LCS bioceramic microspheres. The morphology of LCS microspheres was observed by SEM (SU-8200, Hitachi, Japan), and the size distribution was calculated using Image J software (NIH, USA). XRD (Rigaku D/Max-2550 V, Geigerflex, Japan) was used to determine the phase of LCS microspheres.

Preparation and characterization of GG-LCS bioinks

Bioinks consist of gelatin (Sigma Aldrich, USA), GelMA, and LCS microspheres. GelMA was synthesized through conjugating methacrylate groups into gelatin networks. 2 % (w/v) gelatin and 4 % (w/v) GelMA were dissolved in deionized water containing 0.25 % (w/v) lithium phenyl-2, 4, 6-trimethylbenzoylphosphinate (LAP, Sigma Aldrich, USA) at 50 ℃ for 20 min and then sterilized through a filter (0.22 μm, Millpore, USA). Different contents of sterilized LCS microspheres were added into the hydrogels to form inorganic-organic hybrid bioinks. According to the mass percentage of LCS microspheres to GelMA, the bioinks were named GG, GG-2LCS, GG-5LCS, and GG-10LCS, respectively.

The microstructure of bioinks was observed by SEM (SU-8200, Hitachi, Japan). The rheological behaviors of bioinks were characterized by a rheometer (MCR301, Anton-Paar, Austria). Shear-thinning properties of the bioinks were measured with the shear rate between 1 to 100 s^{-1} at 20 ℃. The storage modulus (G’) and loss modulus

(G'') of the bioinks were tested with the frequency range of 0.1-10 Hz at 20 ℃. For swelling ratio measurement, the lyophilized hydrogels bioinks (*Md*) were firstly placed in PBS for 24 h at 37 ℃ until the weight was no longer changed. Then, the wet weight of the hydrogels (*Mw*) was measured after removing the free water. The swell ratio was calculated according to the formula below:

$$Swelling\ ratio = \frac{Mw - Md}{Md} \times 100\%$$

Mw and *Md* represent the wet and dry weights of the hydrogel bioinks, respectively.

Cell culture

NSCs were isolated from the telencephalons of newborn SD rats (1~2 days old). NSCs were cultured in DMEM/F12 medium (Gibco, USA) supplemented with 2 % (v/v) B-27 (Gibco, USA), 20 ng/mL epidermal growth factor (EGF, PeproTech, USA), 20 ng/mL basic fibroblastic growth factor (bFGF, PeproTech, USA) and 1 % (v/v) penicillin-streptomycin (P/S, Gibco, USA). Besides, for the neural differentiation assay, NSCs were cultured in a differentiation medium containing DMEM/F12 medium, 2 % B-27, 1 % fetal bovine serum (FBS, Gibco, USA), and 1 % P/S. Human umbilical vein endothelial cells (HUVECs) at the passage of 4~8 were used in this study, which were cultured in an endothelial cell medium (Sciencell, USA) supplemented with 2.5 % FBS, 1 % (v/v) endothelial cell growth factor/heparin kit (ECGS/H, Sciencell, USA) and 1% P/S. Rat BMSCs between the passage of 2 to 5 were used and cultured in MEM-α (Gibco, USA) medium with the supplement of 10 % FBS and 1 % P/S. L6 Rat muscle cells, purchased from Cell Bank, Chinese Academy of Sciences, were cultured in high-glucose DMEM medium supplemented with 10 % FBS and 1 % P/S. All cells were cultured in a 37 ℃ humidified incubator with 5 % CO_2.

3D bioprinting of neural constructs

In this work, 3D neural constructs were prepared by a 3D printer (Bioscaffolder 3.2, GeSim, Germany). NSCs with a density of 5×10^6/mL were added into GG hydrogels

and mixed uniformly to form cell-laden bioinks, and then loaded into a syringe and stored at 4 ℃ for 15 min to pre-gelation. Then, the syringe was installed on the 3D printer and connected to extrusion needles (27G). The extrusion pressure was set at the range of 20~50 kPa. The temperature of the syringe was kept at 20 ℃, while the deposition platform was set at 4 ℃. After finishing the printing procedures, the neural construct was further crosslinked by blue light (EFL-LS-1601, EFL, China) for 15 s. Finally, the neural constructs were transferred in a 24-well plate with 1 mL culture medium supplemented and placed into incubator. For the *in vitro* and subcutaneous implantation experiments, the bioinks were deposited in a grid pattern. The height of the constructs was set as 1 mm, the strand distance of filaments was kept at 1.2 mm, and the diameter of the constructs was 8 mm. For the *in vivo* bone defects experiments, the printing parameter was set below: height = 1 mm, diameter = 5 mm, and strand distance = 0.4 mm. For the spinal cord injury and volumetric muscle loss experiments, the bioinks were deposited in a parallel pattern. The size of the neural constructs for spinal cord injury therapy was 3 mm × 3 mm × 3 mm (length × width × height), and for volumetric muscle loss assay was 10 mm × 7 mm × 4 mm (length × width × height), respectively.

Live/dead staining

The survival of cells in LCS-containing medium and 3D bioprinted constructs was assessed by the live/dead staining assay *via* the Calcein-AM/PI kits (Dojindo, Japan). The working solution was prepared as the volume ratio of culture medium: Calcein-AM: PI = 1000: 2: 3. Samples were immersed in the working solution and incubated for 20 min at 37 ℃. Finally, the fluorescent images were captured by a fluorescence microscope (DMi8 S, Leica, Germany).

Cell morphology observation

The morphology of cells in LCS-containing medium and 3D bioprinted constructs were characterized. The samples were fixed in 4 % paraformaldehyde for 30 min, followed by 3 times PBS washing. Then, 0.1 % Triton-X 100 solution was used to

permeabilize the cell membranes. After that, the cytoskeleton and nuclei of cells were labeled with phalloidin (Thermo, USA) and DAPI (Thermo, USA), respectively. Finally, the samples were observed by confocal laser scanning microscopy (CLSM, TCS SP8, Leica, Germany).

RT-qPCR

The gene expression of NSCs in LCS-containing medium and 3D bioprinted constructs was investigated *via* RT-qPCR assay. Briefly, the cell samples were treated with Trizol reagent (Invitrogen, USA) to extract the total RNA. Then, the RNA was reversed into cDNA through the PrimeScript 1st Strand cDNA synthesis kit (TOYOBO, Japan). Subsequently, the gene expression was analyzed in the StepOnePlus Real Time Systems by using SYBR Green QPCR Master Mix reagent (TaKaRa, Japan). GAPDH was set as the housekeeping gene. The relative expression levels of genes were calculated by a $2^{-\Delta\Delta Ct}$ method. All primer sequences of the genes were summarized in Supplementary Table S3.

For 3D bioprinted constructs, the constructs were firstly treated by the GelMA lysis kits (EFL-GM-LS-001, EFL, China) for 30 min to digest the GelMA hydrogels to separate cells within the constructs. Then, the cells were collected through centrifugation. After that, the remaining steps are consistent with the above procedures.

Immunofluorescence protein staining

The relative protein expression level was assessed by immunofluorescence staining assay and analyzed by Image J. Briefly, cell, and 3D bioprinted constructs were fixed in 4 % paraformaldehyde and permeabilized in 0.1 % Triton-X 100 solution. Subsequently, the samples were blocked in 5 % BSA for 1 h to avoid non-specific bindings. After that, primary antibodies were added overnight at 4 ℃, followed by washing with PBS. Finally, a secondary antibody was added to immerse the samples for 1 h. The cell nuclei were stained by DAPI solution. The images were obtained under CLSM and analyzed by Image J. The primary antibodies used in this work were

summarized as below: Nestin (1:200, sc23927, Santa Cruz, USA), GFAP (1:300, ab4674, Abcam, USA), β-III Tubulin (1:300, ab18207, Abcam, USA), MAP2 (1:500, ab5392, Abcam, USA), Neurofilament (1:500, CH22104, Neuromics, USA), CGRP (1:300, ab81887, Abcam, USA), CD31 (1:500, sc-376764, Santa Cruz, USA), VEGF (1:300, ab2349, Abcam, USA), BSP (1:300, ab226243, Abcam, USA), OCN (1:500, sc-390877, Santa Cruz, USA), MHC (1:500, ab37484, Abcam, USA) and AchRs (1:200, sc-65829, Santa Cruz, USA).

(5-ethynyl-2' -deoxyuridine) EdU staining assay

The proliferation activity of NSCs within the 3D bioprinted constructs was evaluated *via* EdU cell proliferation kits (C0071S, Beyotime, China). Briefly, the 3D bioprinted constructs were incubated with EdU working solution for 24 h at 37 ℃. Then, the samples were fixed in 4 % paraformaldehyde. Cell nuclei were stained with DAPI for 10 min. Finally, the images were captured by CLSM and analyzed by Image J.

Western blot assay

The chemical inhibitors LY294002 with a concentration of 25 μM (9901, CST, USA) were applied to inhibit the PI3K-AKT signaling pathway. After 48 h culture, NSCs were firstly separated from the 3D bioprinted constructs and then lysed by RIPA buffer (PC101, Epizyme Biotech, China) containing 1 % phosphatase inhibitors (GRF102, Epizyme Biotech, China) and 1 % protease inhibitors (GRF101, Epizyme Biotech, China). The extracted proteins were measured by a BCA protein assay kit (ZJ102, Epizyme Biotech, China). Proteins were separated *via* SDS-PAGE and then transferred into PVDF membranes (Millipore, USA). Subsequently, the membranes were blocked by 10 % BSA for 1 h and incubated with primary antibodies overnight at 4 ℃. Then the membranes were incubated with IRDye 800CW Goat anti-Rabbit IgG secondary antibodies (926-32211, LI-COR, USA) for 1 h and observed by the Odyssey® DLx (LI-COR, USA) system. The primary antibodies used in this work were summarized as follows: PI3K (1:500, 4292, CST, USA), p-PI3K (1:500, 4228, CST, USA), p-AKT (1:500, 4060, CST, US), AKT (1:500, 4691, CST, US), GSK-3β

(1:500, 12456, CST, USA), β-III Tubulin (1:300, ab18207, Abcam, USA), and GAPDH (AC001, Abclonal, China).

RNA Sequencing

NSCs were separated from the neural constructs after being cultured in the differentiation medium for 10 days. Followed by extracting the total RNA. The subsequent RNA sequencing process was conducted by Shanghai Biotechnology Corporation according to their protocol. Differentially expressed genes (DEGs) were identified by a cutoff: p-value < 0.05 and $|\log_2$ Fold Change$| > 1$.

Enzyme-like immunosorbent assay (ELISA) assay

The supernatant of the medium of 3D bioprinted neural constructs was harvested after 2 and 4 days of culture. The secretion of calcitonin gene-related peptide (CGRP) and nerve growth factor (NGF) from the neural constructs were detected by the CGRP kits (E-EL-R0135c, Elabscience, China) and NGF kits (E-EL-R0652c, Elabscience, China). The final data were processed according to the manufacturer's protocols.

Modulatory effects of 3D bioprinted neural constructs on osteogenesis

To evaluate the modulation effects of 3D bioprinted constructs on osteogenesis, BMSCs were indirectly co-cultured with 3D bioprinted neural constructs using a transwell chamber. The co-culture medium was prepared by mixing the DMEM/F-12 differentiation medium and MEM-α medium with a volume ratio of 1:1.

For the BMSCs migration assay, the transwell chambers (8 μm, Corning, USA) were used. BMSCs were seeded in the upper chamber, and the four kinds of 3D printed constructs were prepared and placed in the bottom of a 24-well plate. After incubating in a serum-free medium for 12 hours, the chamber was fixed in 4 % paraformaldehyde, and the non-migrated cells were gently removed. The migrated cells were stained with 0.1 % crystal violet solution for 1 min. The images were observed by a microscope, and the migrated cell numbers were counted by Image J software.

For the immunofluorescence protein staining assay, 3D bioprinted constructs were placed on the upper chamber (0.4 μm, Corning, USA), and BMSCs were seeded in the bottom. After being co-cultured for 5 days, 3D printed constructs within the upper chamber were removed, and the subsequent procedures are consistent with the immunofluorescence protein staining section mentioned above. After being co-cultured for 7 days, BMSCs were fixed in 4 % paraformaldehyde, followed by being immersed in an ALP staining solution (C3250S, Beyotime, China) for 30 min. The images were observed by microscope. ALP activity was detected by the kits (P0321S, Beyotime, China). After co-cultured for 10 days, BMSCs were fixed in 4 % paraformaldehyde, followed by stained with Alizarin Red S staining solution (C0138, 2 %, pH = 4.2, Beyotime, China) for 30 min at 37 ℃. Cell samples were observed by microscope. The quantitative analysis was determined in a microplate reader at the 405 nm wavelength.

Modulatory effects of 3D bioprinted neural constructs on angiogenesis

To evaluate the modulation effects of 3D bioprinted constructs on angiogenesis, HUVECs were indirectly co-cultured with 3D bioprinted neural constructs using a transwell chamber. The co-culture medium was prepared by mixing the DMEM/F-12 differentiation medium and ECM medium with a volume ratio of 1: 1. For the HUVEC migration assay, HUVECs were seeded in the upper chamber (8 μm pore), and the constructs were placed in the bottom. After 12 h of incubation with a serum-free co-culture medium, the chamber was fixed in 4 % paraformaldehyde, and the migrated cells were stained with 0.1 % crystal violet solution. For tube formation assay, HUVECs were seeded on Matrigel (354262, Corning, USA), and constructs were placed in the upper chamber and then co-cultured for 6 h. Then, the tube formation capacity of HUVECs in each group was assessed by microscope. The number of junctions and meshes in each field were further analyzed by Image J. After being co-cultured for 5 days, HUVECs were fixed for immunofluorescence protein staining experiments. The expression of CD31 and VEGF were detected, and the procedures were consistent, as mentioned above.

In vitro formation of neuromuscular junctions (NMJs)

Double immunofluorescence staining experiments were assessed to determine the capacity of 3D bioprinted constructs for forming neuromuscular junctions *in vitro*. Briefly, 3D bioprinted GG-NSC and GG-LCS-NSC constructs were first prepared *via* the 3D printer. Then L6 rat muscle cells were directly seeded onto the constructs and co-culture for 5 days. The co-culture medium was prepared by mixing the DMEM/F-12 differentiation medium and high glucose DMEM medium with a volume ratio of 1: 1. At the time point, double-immunofluorescence of rabbit anti-β-III Tubulin/rat anti-AChR antibodies were applied to evaluate the NMJs formation *in vitro*. Nuclei were stained by DAPI. The images were observed in CLSM, and NMJs numbers per field were counted by Image J software.

Subcutaneous implantation experiments

All the animal experiments were conducted under the guidelines of the Institutional Animal Care and Utilization Committee of Nanjing First Hospital, Nanjing Medical University (DWSY-22030156). For subcutaneous implantation assay, SD rats (6-8 weeks old, 200-220 g) were purchased from Shanghai Lab Animal Research Center, China. NSCs were labeled by green fluorescent protein (GFP) before being incorporated into bioinks. Then, the neural constructs were prepared and cultured for 7 days before implantation. Rats were anesthetized, and the dorsal hair was removed. Then, the 3D bioprinted neural constructs were implanted into the subcutaneous cavity of the back. After 7 and 14 days of post-implantation, the constructs were collected and fixed in 4 % paraformaldehyde for further histological analysis.

Spinal cord transection model and functional recovery analysis

A complete spinal cord transection model was executed to assess the potential of neural constructs for repairing central nervous system injury. Female SD rats with a weight range of 220-250 g were used to create transection defects. All the constructs were prepared (3 mm × 3 mm × 3 mm, length × width × height) and cultured for 7

days before implantation. Briefly, rats were anesthetized, and the back skin was incised to expose the T9-T11 vertebrae. After the resection of vertebrae, the complete transection of the spinal cord with a length of approximately 3 mm was made. Commercial gelatin sponges were used to control the bleeding. Then, the 3D bioprinted constructs were implanted into the 3 mm transected gap, followed by suturing the skin. After surgery, the bladder was manually massaged twice in the morning and evening until it automatically restored the urination function.

The locomotor function recovery was assessed every week under the guidelines of Basso, Beattie and Bresnahan (BBB) locomotor ability scale. Rats were allowed to move in a free space. The hindlimb movement of rats was recorded by blinded observers, with the BBB score ranging from 0 (complete paralysis) to 21 (normal movement).

Electrophysiological analysis was performed after 8 weeks post-surgery to evaluate the neural conduction functions. After anesthesia, the stimulating electrodes were placed on the surface of the brain, corresponding to the motor area of the cerebral cortex. Then, the recording electrodes were inserted into the tibialis anterior muscle of the contralateral hind limb to record the motor-evoked potentials (MEPs).

Cranial bone defects model and radiological analysis

A cranial bone defects model was established to assess the ability of 3D bioprinted neural constructs to promote bone formation and bone innervation. Briefly, 8-week-old male SD rats with a weight in the range of 220-250 g were applied to create critical bone defects with a diameter of 5 mm. All the constructs were fabricated with a diameter of 5 mm and height of 1 mm, and cultured for 7 days before implantation. After 8 weeks of implantation, rats were sacrificed to harvest the cranial bone samples for further analysis.

After being fixed for 48 h, all skull tissues were scanned by the micro-computed tomography system (Micro-CT, SKYSCAN1172, Bruker, Germany) with a resolution of 8.9 μm. The values of bone volume/total volume (BV/TV), bone mineral density (BMD), trabecular number (Tb.N), and trabecular separation/spacing (Tb.Sp) were

determined by the CT-Analyzer software (Bruker, Germany). Subsequently, the bone samples were decalcified with 10 % EDTA solution (G1105, Servicebio, China) for further histological analysis.

VML defect model experiments

Critical tibialis anterior (TA) muscle defect model was established to assess the ability of neural constructs to promote skeletal muscle regeneration and innervation. 8-weeks-old male SD rats with a weight range of 220-250 g were applied to create TA defects. All the constructs were prepared (10 mm × 7 mm × 4 mm, length × width × height) and cultured for 7 days before implantation. Briefly, rats were anesthetized, and then the skin of the lower left leg was incised to expose TA muscle. According to the following formulas: W (g) = 0.0017 × body weight (g) – 0.0716, approximately 40 % of TA muscle was excised and weighted. Then, 3D bioprinted constructs were implanted into the defects and covered with skin. After 8 weeks of post-implantation, TA muscles of the lower left leg were weighted, and then their percentage of contralateral muscles was calculated.

Histological analysis

All the samples collected from the animal experiments were dehydrated overnight with 10 % and 30 % sucrose solution, then embedded in an optimal cutting temperature (OCT) compound. Subsequently, collected tissues were sectioned into 10 μm slices by a freezing microtome (CryoStar NX70, Thermo, USA).

For H&E and Masson’s trichrome staining, the slices were first immersed in a PBS solution for 20 min to remove the residue OCT compounds. Then the slices were stained with an H&E staining kit (C0105S, Beyotime, China) and Masson’s trichrome staining kit (G1340, Solarbio, China), respectively. The images were obtained by an optical microscope (Zeiss, Germany).

For the immunohistochemistry staining assay, the slices were soaked in PBS solution, followed by immersed in proteinase K working solution (P78893, Abcone, China) for antigen retrieval. Then, the slices were blocked in 10 % donkey serum

(BL939A, Biosharp, China) and 0.3 % TritonX-100 (Sigma Aldrich, USA) for 1 h at room temperature. Next, the slices were incubated with primary antibodies overnight at 4 ℃, and subsequently incubated with secondary antibodies for 1 h at room temperature. Finally, the slices were stained with DAPI for 10 min and covered with fluorescent mounting medium (S3023, Dako, Denmark). The immunofluorescent images were captured with CLSM and analyzed by Image J.

Statistics analysis

All data were expressed as mean ± standard deviation (SD) and repeated at least 3 times independently with similar results. All the statistical analysis was performed using Origin 2021 software (Origin Lab, USA). Significant differences were calculated using one-way analysis of variance (ANOVA) with post-hoc Tukey tests. Significant differences were considered when the p value less than 0.05 ($*p < 0.05$; $**p < 0.01$; $***p < 0.001$).

Supplementary Figures and Tables

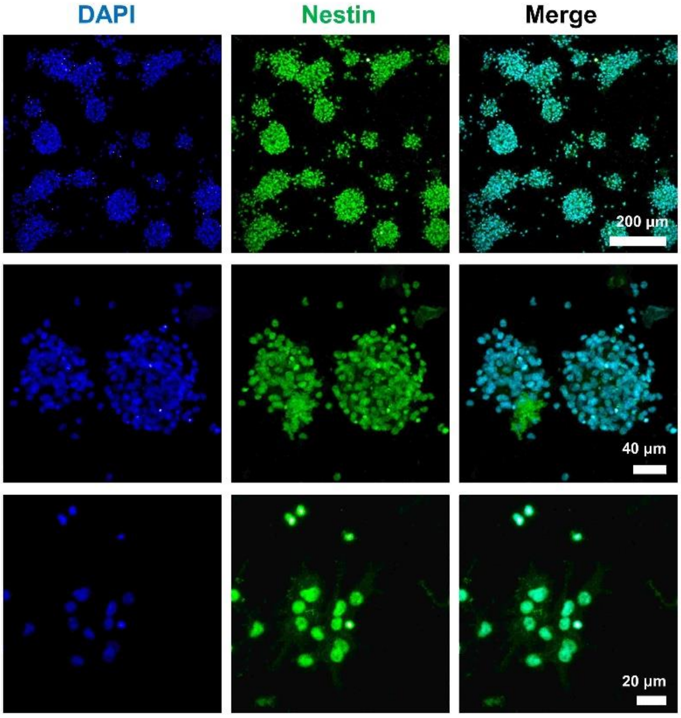

Figure S1. Immunofluorescence staining of stemness marker Nestin of the isolated NSCs.

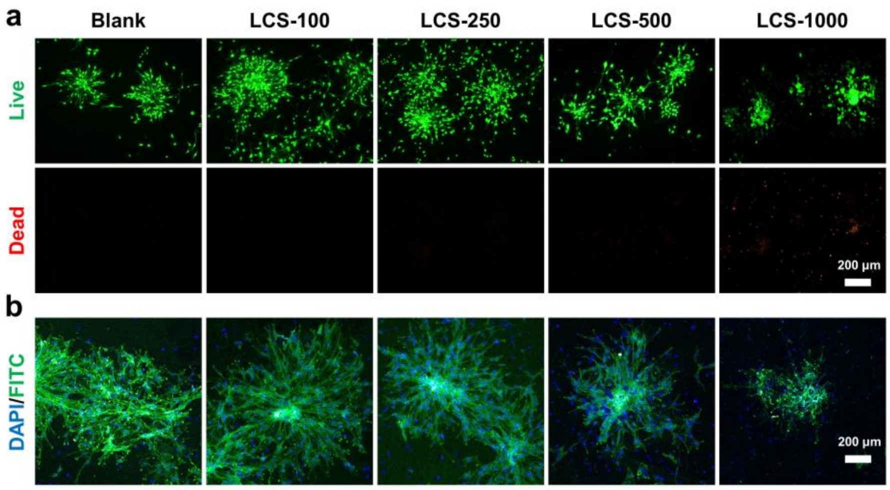

Figure S2. Live/dead staining (a) and morphology observation (b) of NSCs after culture with LCS microspheres.

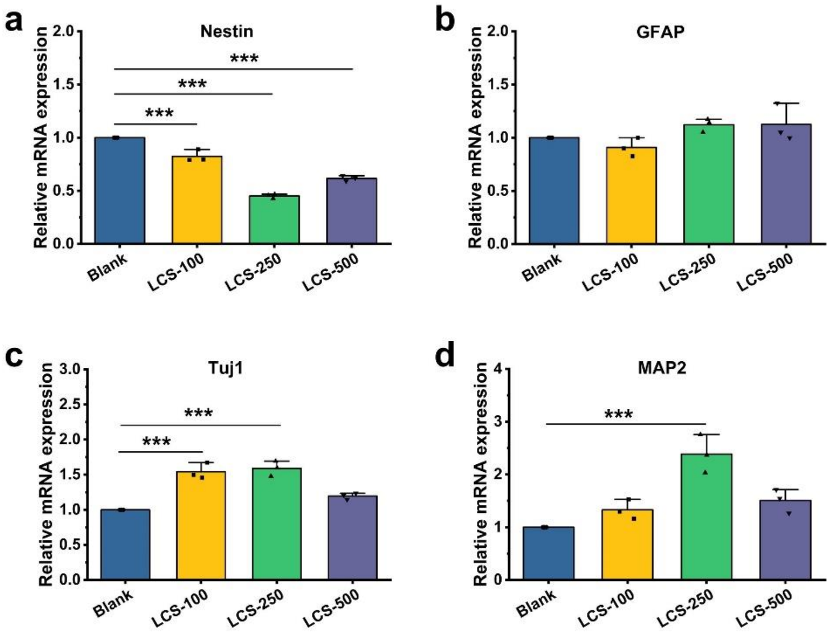

Figure S3. RT-qPCR analysis of the relative mRNA expression level of stemness marker Nestin (a), astrocytes marker glial fibrillary acid protein (GFAP) (b), neuron marker β-III tubulin (Tuj1) (c) and mature neurons marker microtubule-associated protein 2 (MAP2) (d) in NSCs after 5 days of culture with LCS microspheres (n = 3). Data are presented as the mean value ± SD. *$p < 0.05$, **$p < 0.01$, ***$p < 0.001$.

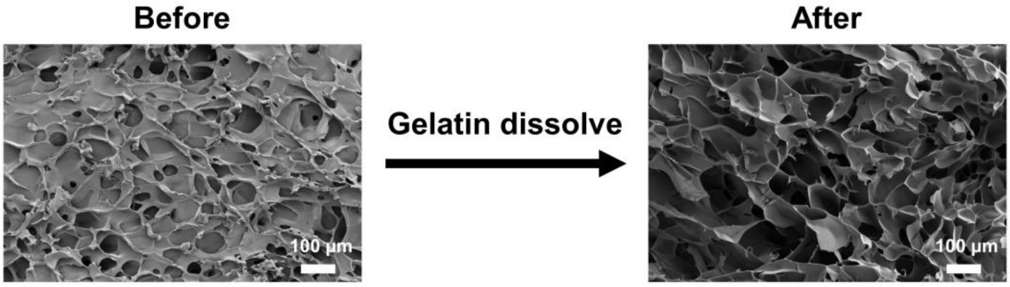

Figure S4. The SEM images of the GG bioinks before and after the gelatin dissolved.

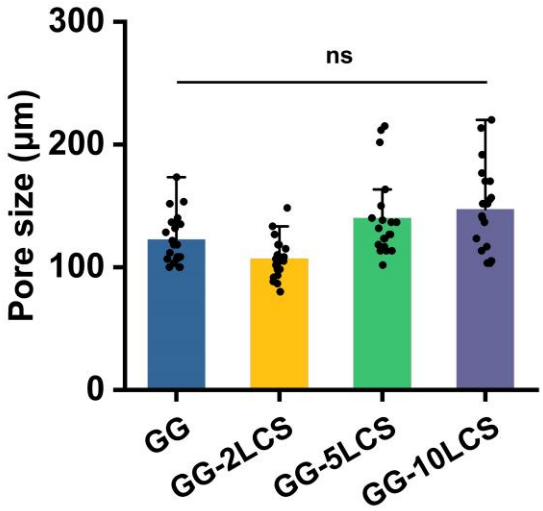

Figure S5. The pore size distribution of the bioinks (n = 20). n.s represents no significant difference.

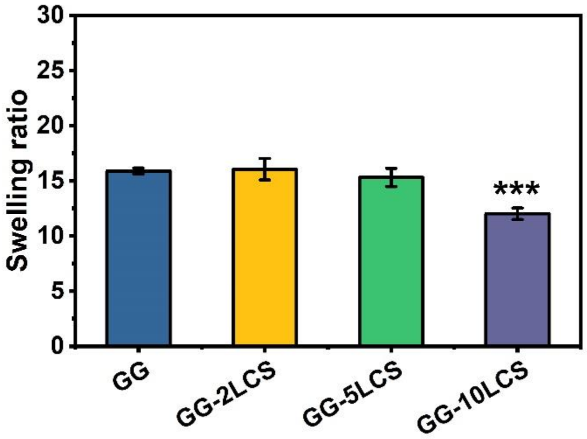

Figure S6. The swelling ratio properties of the bioinks. Data are presented as the mean value ± SD. *$p < 0.05$, **$p < 0.01$, ***$p < 0.001$.

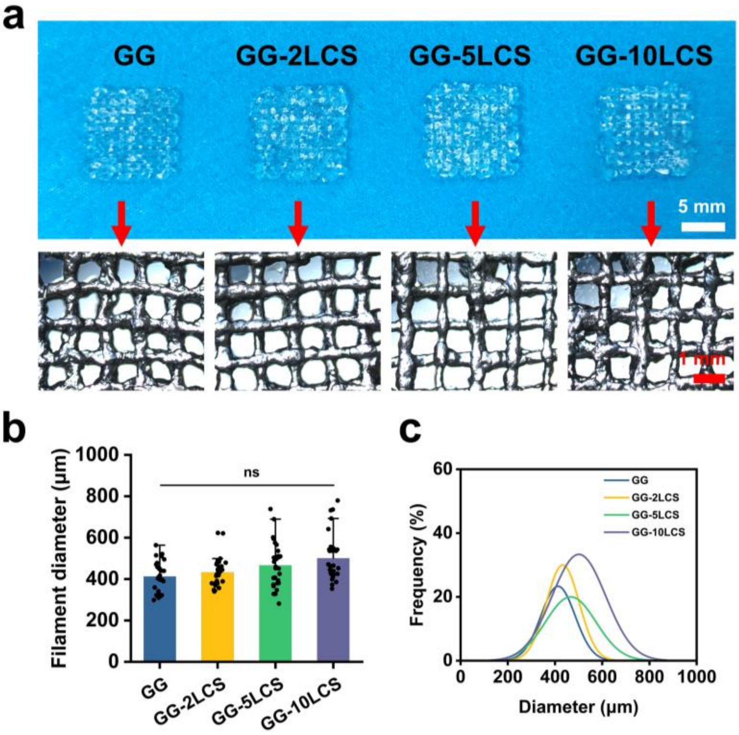

Figure S7. (a) Photographs of the 3D bioprinted GG, GG-2LCS, GG-5LCS and GG-10LCS constructs. (b-c) Filament diameter distribution of these constructs. n.s represents no significant difference. n.s represents no significant difference.

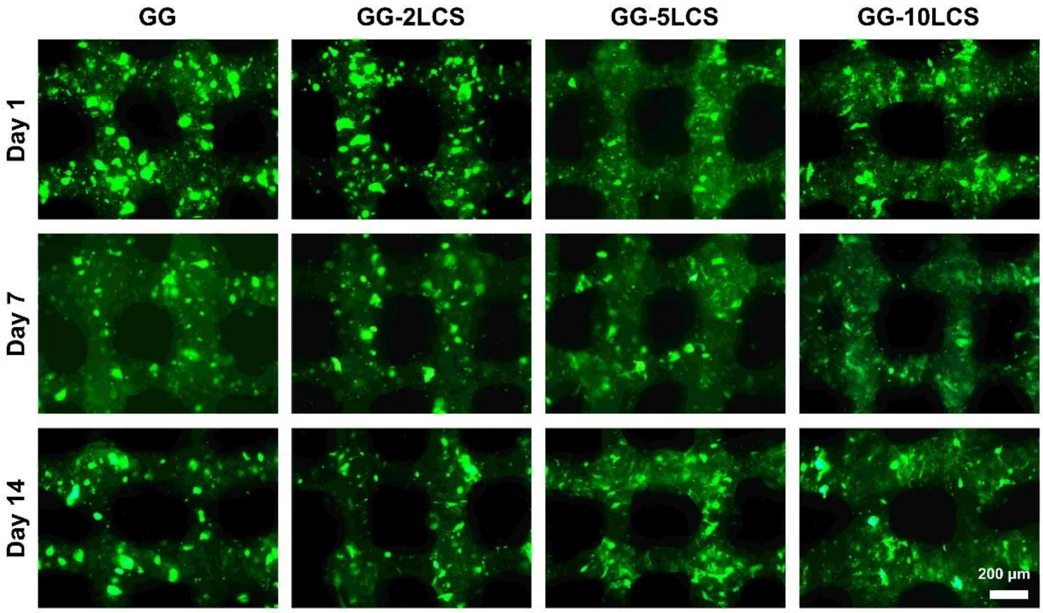

Figure S8. The live/dead staining images of the 3D bioprinted neural constructs after cultured for 1, 7, and 14 days.

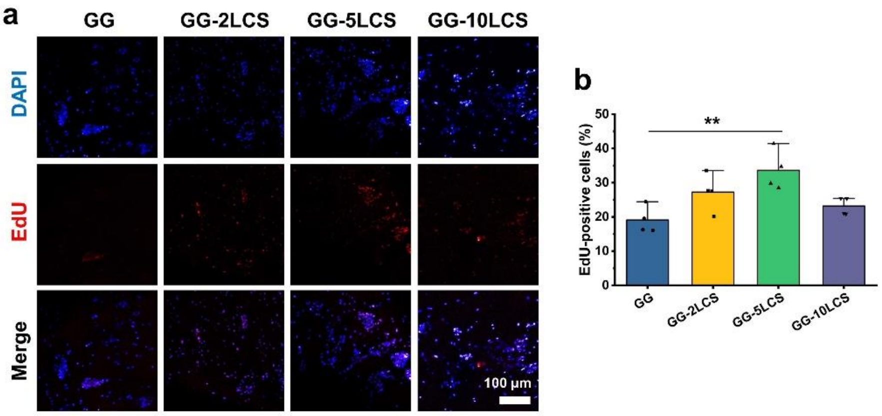

Figure S9. (a) Representative EdU staining images of the bioprinted constructs after culture in proliferation medium for 2 days. (b) The quantification percentage (normalized to nuclei) of EdU-positive cells per field (n = 4). Data are presented as the mean value ± SD. *p < 0.05, **p < 0.01, ***p < 0.001.

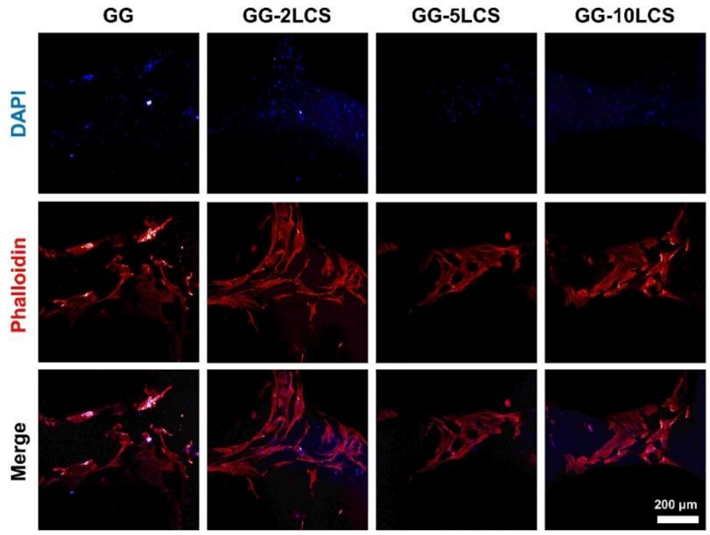

Figure S10. The morphology of NSCs within the bioprinted constructs after 10 days of culture.

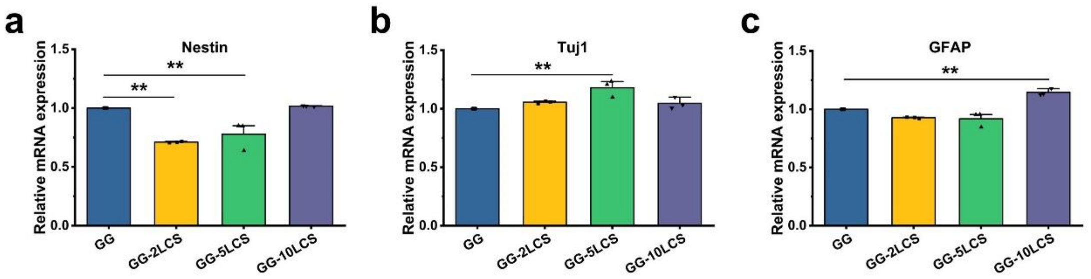

Figure S11. The relative mRNA expression of Nestin (**a**), Tuj1 (**b**), and GFAP (**c**) of NSC within these bioprinted constructs after 10 days of culture in differentiation medium (n = 3). Data are presented as the mean value ± SD. *$p < 0.05$, **$p < 0.01$, ***$p < 0.001$.

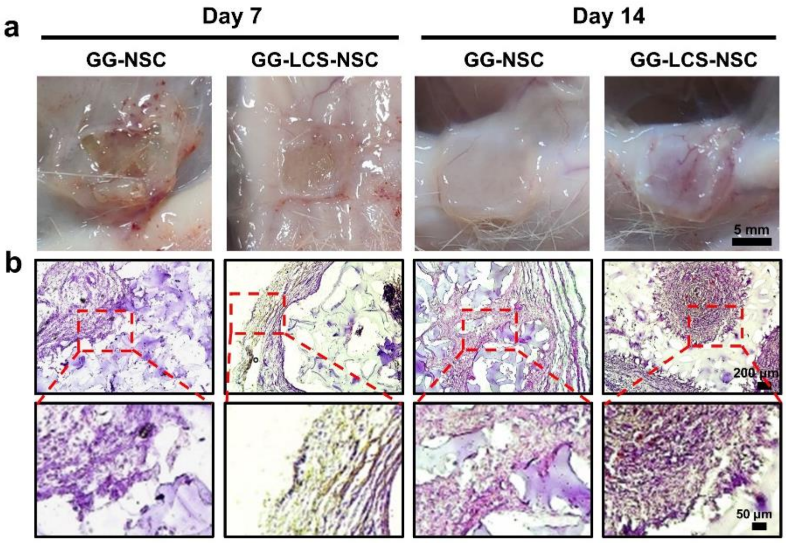

Figure S12. (a) The optical images of the GG-NSC and GG-LCS-NSC constructs after 7 and 14 days of post-implantation. (b) Hematoxylin & eosin (H&E) staining images of the implanted constructs at days 7 and 14.

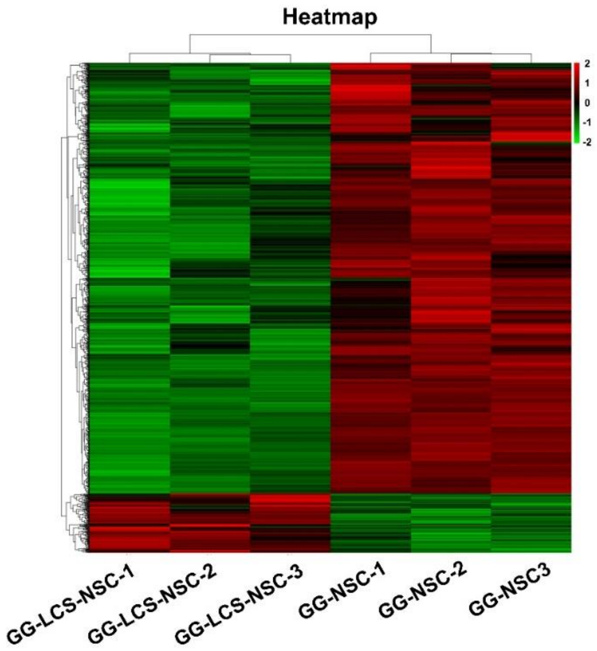

Figure S13. The heatmap of the differentially expressed genes between GG-NSC and GG-LCS-NSC groups. Up-regulated genes were marked in red, while down-regulated genes were marked in green.

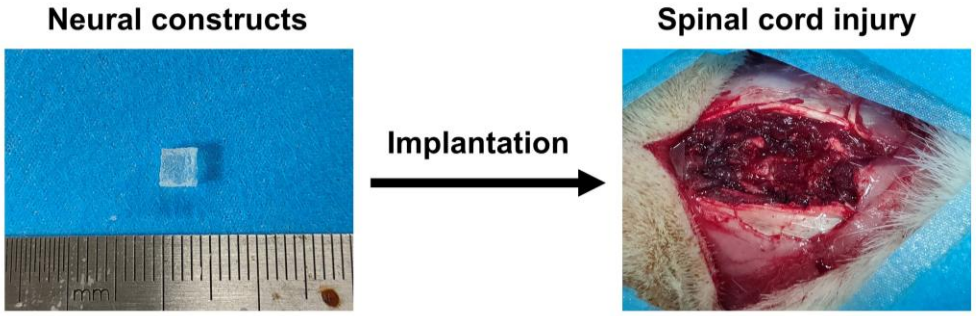

Figure S14. Gross images of the neural constructs and the implantation of neural constructs into the lesion area of SCI rats.

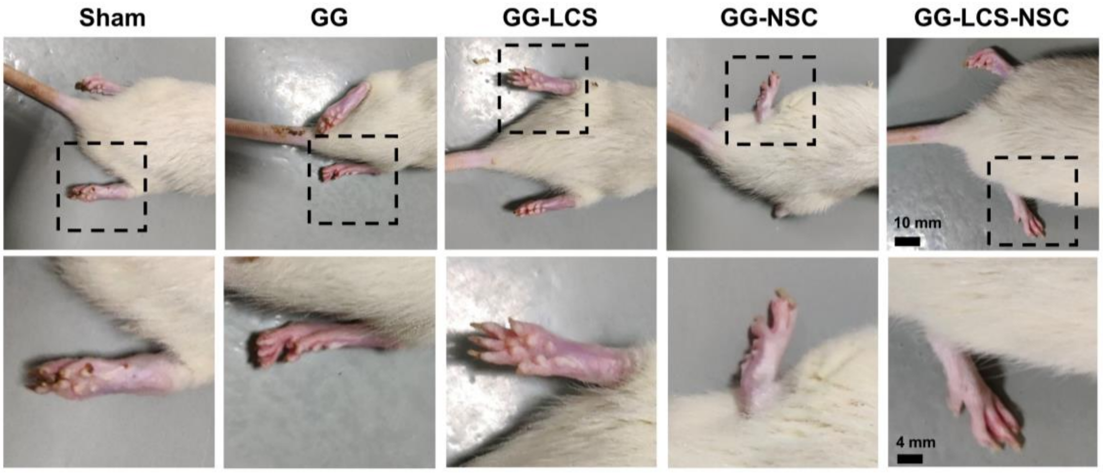

Figure S15. The representative images of the hindlimbs-spreading status of SCI rats after 8 weeks of post-surgery.

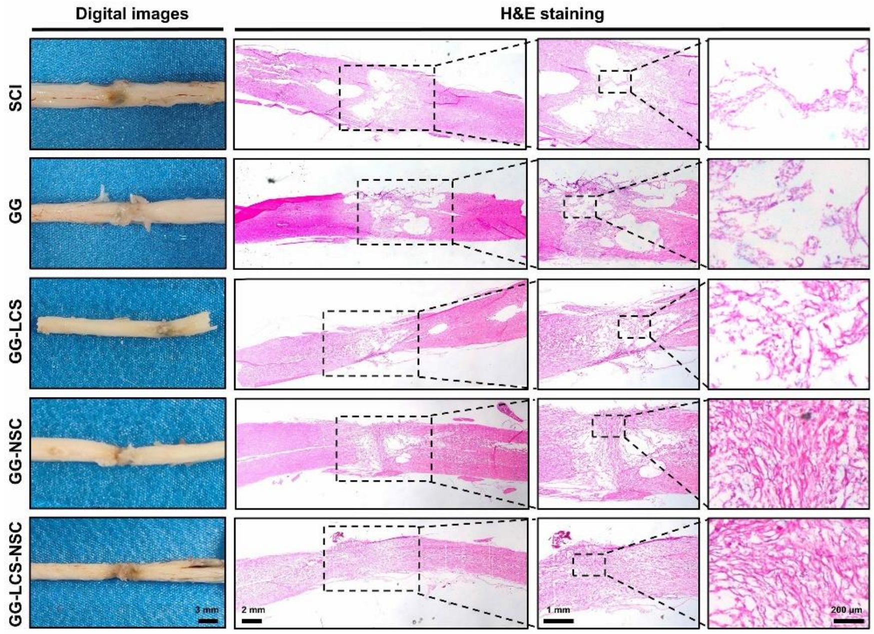

Figure S16. The optical images and H&E staining images of the harvested spinal cords after 8 weeks of post-surgery.

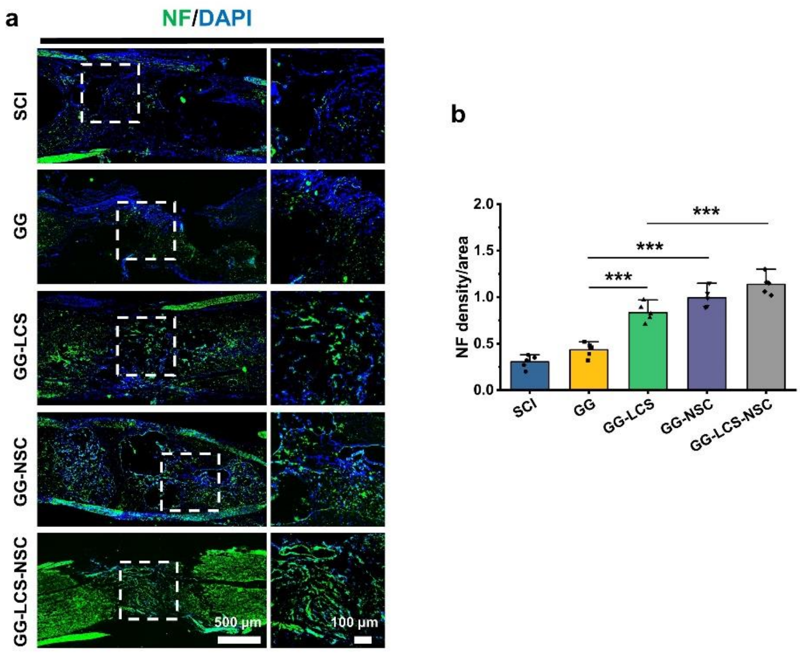

Figure S17. (a) Representative immunofluorescence staining images of NF of the longitudinal sections of spinal cords to evaluate the neuronal regeneration. (b) Statistical analysis of the NF (g), positive areas in the lesion regions (n = 5). Data are presented as the mean value ± SD. *p < 0.05, **p < 0.01, ***p < 0.001.

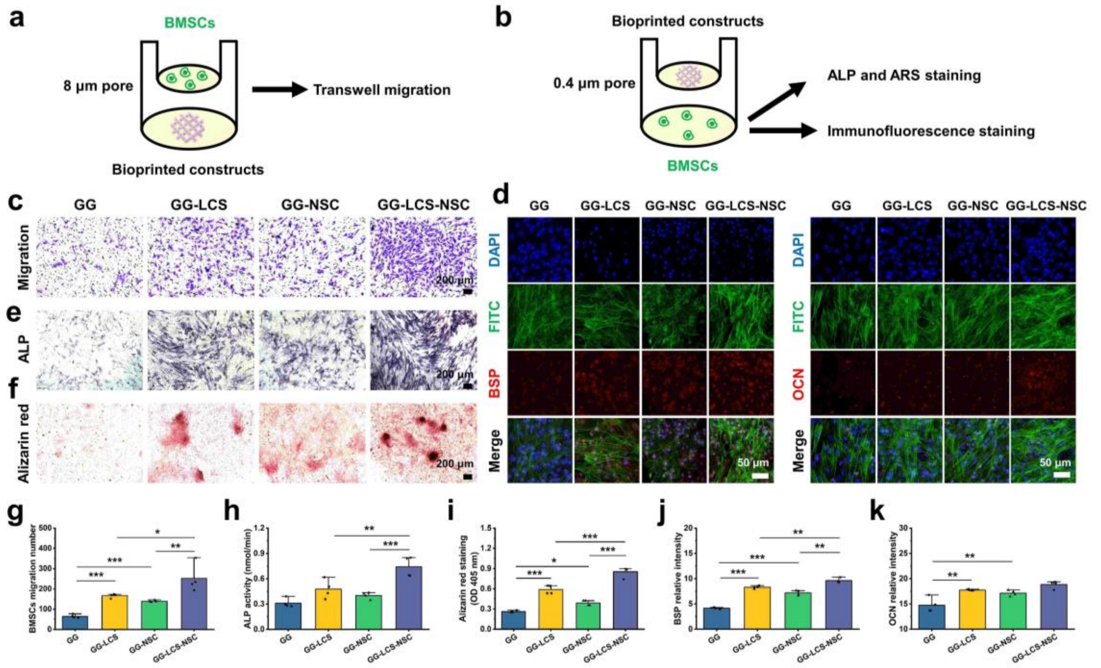

Figure S18. In vitro assessment of 3D bioprinted neural constructs on osteogenesis. (a-b) Schematic depiction of the indirect co-culture of BMSCs and bioprinted constructs for transwell migration assay (a), immunofluorescence staining, alkaline phosphatase (ALP) staining, and Alizarin red S (ARS) staining assay (b). (c) Typical bright-field images of the migrated BMSCs (stained with crystal violet) in each group. (d) Representative images of the osteogenic marker BSP (red) and OCN (red) of BMSCs after 5 days of co-culture with different constructs. (e) Typical microscope images of the ALP staining of BMSCs after co-culture for 7 days. (f) Representative ARS staining images of BMSCs after 10 days of co-culture. (g) Quantitative analysis of the migrated BMSCs in each group (n = 4). (h-i) Statistical analysis of the ALP activity (h) and OD values of calcium nodules of BMSCs (i) (n = 4). (j-k) Quantitative analysis of BSP (j) and OCN (k) proteins of BMSCs with different treatments (n = 4). Data are presented as the mean value ± SD. $*p < 0.05$, $**p < 0.01$, $***p < 0.001$.

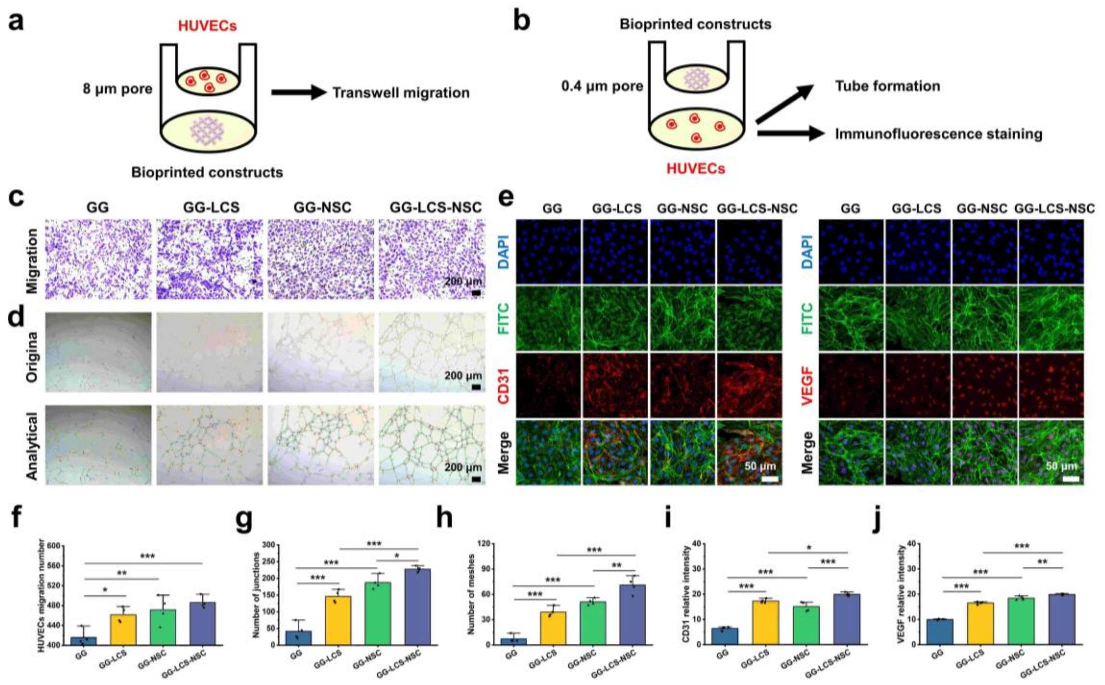

Figure S19. In vitro assessment of 3D bioprinted neural constructs on angiogenesis. (a-b) Schematic depiction of the indirect co-culture of HUVECs and bioprinted constructs for transwell migration assay (a), tube formation, and immunofluorescence staining assay (b). (c) Typical bright-field images of the migrated HUVECs (stained with crystal violet) in each group. (d) The original and corresponding analytical images of tube formation assay of HUVECs with different treatments. (e) Representative immunofluorescence staining images of angiogenic marker CD31 (red) and VEGF (red) of HUVECs after 5 days of co-culture with bioprinted constructs. (f) Quantitative analysis of the migrated HUVECs with different treatments (n = 4). (g-h) Statistical analysis of the number of junctions and meshes formed by HUVECs (n = 4). (i-j) Mean fluorescence intensity analysis of CD31 (i) and VEGF (j) proteins of HUVECs (n = 4). Data are presented as the mean value ± SD. *$p < 0.05$, **$p < 0.01$, ***$p < 0.001$.

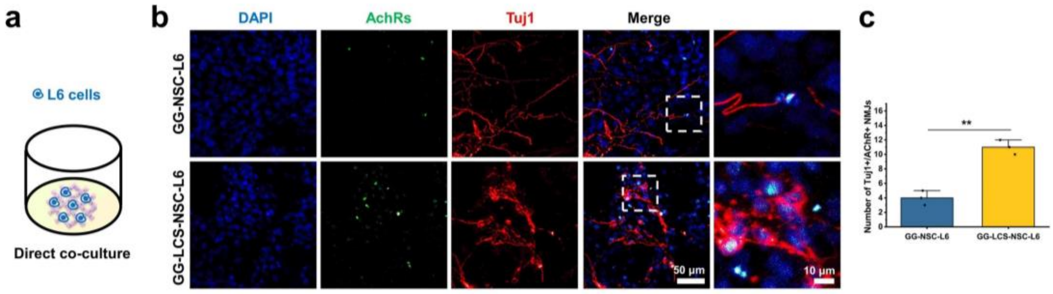

Figure S20. In vitro assessment of 3D bioprinted neural constructs on forming of NMJs. (a) Schematic depiction of the direct co-culture of rat muscle L6 cells and bioprinted constructs. (b) Double-immunofluorescence staining images of AchRs (green) and Tuj1 (red) for the evaluation of NMJs. (c) Quantification of the number of NMJs ($AchRs^+/Tuj1^+$) per field ($n = 3$). Data are presented as the mean value ± SD. $*p < 0.05$, $**p < 0.01$, $***p < 0.001$.

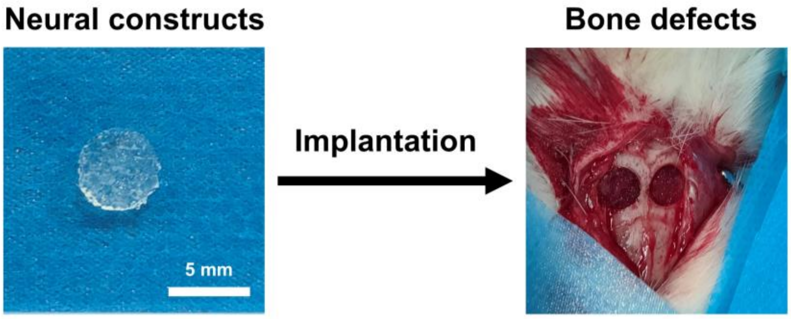

Figure S21. Images of the neural constructs and then implanted into the rat cranial bone defects.

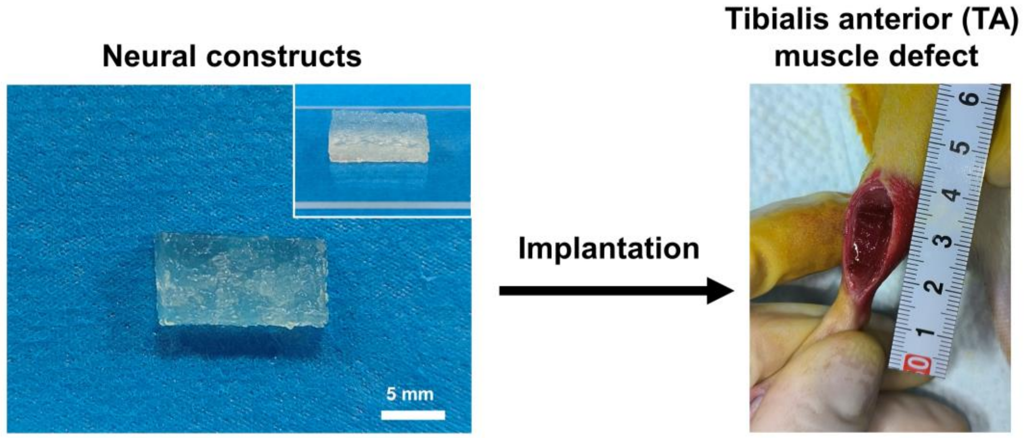

Figure S22. Images of the neural constructs and then implanted into the tibialis anterior (TA) muscle defect.

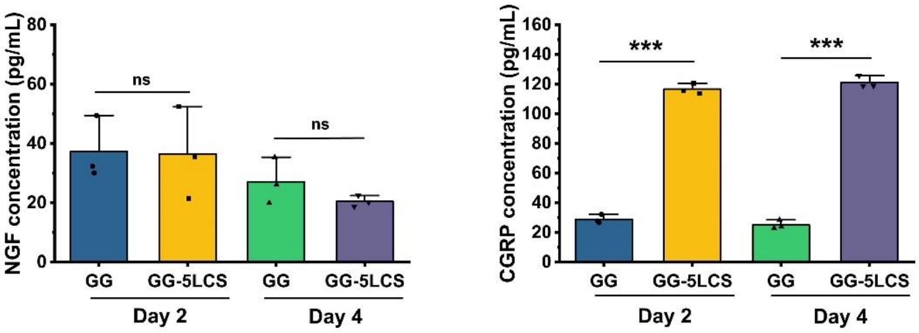

Figure S23. The cytokine release behaviors of NGF and CGRP of the bioprinted neural constructs after 2 and 4 days of culture. Data are presented as the mean value ± SD. *$p < 0.05$, **$p < 0.01$, ***$p < 0.001$.

Table S1. The Ca, Li, and Si ions release behaviors of LCS microspheres after being cultured for 1, 3 and 5 days. (n = 4).

Ions released from the LCS microspheres

**Ionic Conc ($mg\ L^{-1}$)**	**Time (d)**	**LCS-100 μg/mL**	**LCS-250 μg/mL**	**LCS-500 μg/mL**	**LCS-1000 μg/mL**
**Ca**	1	133.65±4.47	201.55±4.60	246.68±6.37	465.63±2.45
	3	81.78±0.62	88.06±0.66	89.16±0.75	127.80±1.20
	5	37.24±0.33	37.43±0.39	35.34±0.85	36.68±0.22
**Li**	1	5.47±0.14	11.87±0.43	22.30±0.68	48.56±0.37
	3	0.07±0.01	0.13±0.00	0.34±0.01	0.83±0.01
	5	0	0	0	0
**Si**	1	39.04±1.82	85.11±1.32	128.31±4.40	137.81±1.74
	3	4.62±0.86	11.52±0.64	45.78±1.29	105.95±2.59
	5	0	0	1.31±0.52	48.44±1.20

Table S2. The Ca, Li, and Si ions release behaviors of the bioprinted neural constructs after being cultured for 1, 4, 7, 10, and 14 days. (*n* = 3).

Ions released from the 3D bioprinted neural constructs

**Ionic Conc (mg L^{-1})**	**Time (d)**	**GG**	**GG-2LCS**	**GG-5LCS**	**GG-10LCS**
**Ca**	1	33.51±0.16	93.93±1.65	146.54±3.92	240.72±1.03
	4	39.34±0.34	52.35±0.56	68.35±1.02	80.93±1.69
	7	41.22±0.10	43.00±0.09	46.65±0.38	47.49±0.29
	10	40.99±0.55	39.74±0.74	40.86±0.47	39.89±0.35
	14	39.62±0.87	39.07±0.3	38.82±0.52	37.38±0.32
**Li**	1	0	4.58±0.79	10.86±2.42	22.78±2.46
	4	0	1.28±1.18	3.2±0.68	5.27±0.66
	7	0	0	0.47±0.82	0.87±0.24
	10	0	0	0	0
	14	0	0	0	0
**Si**	1	0	41.33±0.69	68.68±2.64	80.87±0.98
	4	0	8.45±1.10	26.01±1.22	58.30±0.29
	7	0	0	8.59±1.89	49.95±0.88
	10	0	0	3.69±1.13	26.45±1.16
	14	0	0	0	7.26±1.21

Table S3. The primer sequences used for RT-qPCR assays.

Gene	Primer sequences
GAPDH	5′-AGTGCCAGCCTCGTCTCATA -3′ (forward)
GAPDH	5′-GATGGTGATGGGTTTCCCGT-3′ (reverse)
Nestin	5′-AGCACTCCCATCCCACCTAT-3′ (forward)
Nestin	5′-GGGTTGTGGCTAAGGAGGTC-3′ (reverse)
GFAP	5′-CTTGACCTGCGACCTTGAGT -3′ (forward)
GFAP	5′- TCTTCGCCCTCCAGCAATTT-3′ (reverse)
Tuj1	5′-TCAAGGTAGCGGTGTGTGAC-3′ (forward)
Tuj1	5′-GTGTACCAGTGGAGGAAGGC-3′ (reverse)
MAP2	5′- GTTCAGGCCCACTCTCCTTC-3′ (forward)
MAP2	5′-GGGAGGATGGAGGAAGGTCT-3′ (reverse)
Sgk2	5′-AGTCAGGCGAGTGGGTGACAGG-3′ (forward)
Sgk2	5′-TGGAAACCCCCAGGTTCCAGC-3′ (reverse)
Angpt1	5′-GCTGGCAGTACAATGACAGT-3′ (forward)
Angpt1	5′-GTATCTGGGCCATCTCCGAC-3′ (reverse)
Brca1	5′-TGTCCTTCATGCTATGCAGA-3′ (forward)
Brca1	5′-GCACTTCCTTGTAGGCTCCT-3′ (reverse)
Erbb4	5′-AACTGCACCCAGGGGTGTAA-3′ (forward)
Erbb4	5′-GACATAAACGGCAAATGTCA-3′ (reverse)
Areg	5′- GGTGAATGCAGATACATCGAGA-3′ (forward)
Areg	5′- CGTTCGCCAAAGTAATCCTG-3′ (reverse)
